# Enantioselective Addition of 1,3,5,7-Tetramethyl-BODIPYs to Isatins by Bifunctional Quinine-Based Squaramides

**DOI:** 10.1021/acsomega.4c08792

**Published:** 2025-01-01

**Authors:** Esra Dündar, Murat Işık, Erol Yildirim, Cihangir Tanyeli

**Affiliations:** † Department of Chemistry, Middle East Technical University, 06800 Ankara, Türkiye; ‡ Department of Food Engineering, Bingöl University, 12000 Bingöl, Türkiye

## Abstract

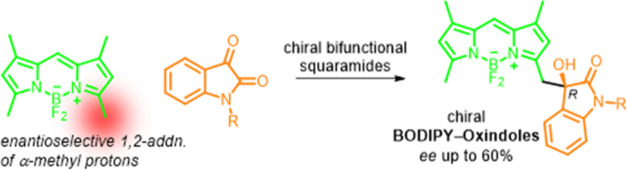

This work describes the development of the first enantioselective addition reaction between 1,3,5,7-tetramethyl-BODIPYs and isatin derivatives. The reaction utilizes bifunctional quinine/squaramide organocatalysts and affords nine novel chiral BODIPY dyes under mild conditions, with enantioselectivities reaching up to 60%. The synthesized BODIPY–oxindoles exhibit high fluorescence emissions, consistent with their parent BODIPYs, and display tunable colors. A representative example demonstrates a remarkably high quantum yield of 0.78 compared to fluorescein. Notably, the newly created carbon-stereocenter on the isatin skeleton induces detectable asymmetry in the electronically decoupled BODIPY chromophore. This is confirmed by the presence of Cotton effects in the visible region of the electronic circular dichroism (ECD) spectra. Density Functional Theory calculations suggested that the model oxindole **3aa** adopts an (*R*) absolute stereochemical configuration, unveiling key interactions between the catalyst and substrates.

## Introduction

Difluoroboron bridged dipyrromethene (BODIPY) dyes, first introduced in 1968, have become increasingly popular due to their exceptional chemical and physical properties.^1−3^ These brightly colored molecules exhibit outstanding spectroscopic features, including high fluorescence quantum yields, large molar absorptivities, narrow emission bands, and minimal triplet states. This unique combination of desirable properties translates to broad applications in various fields, including biological labeling, laser dyes, chemosensors, and indicators.^4^

**Figure 1:**
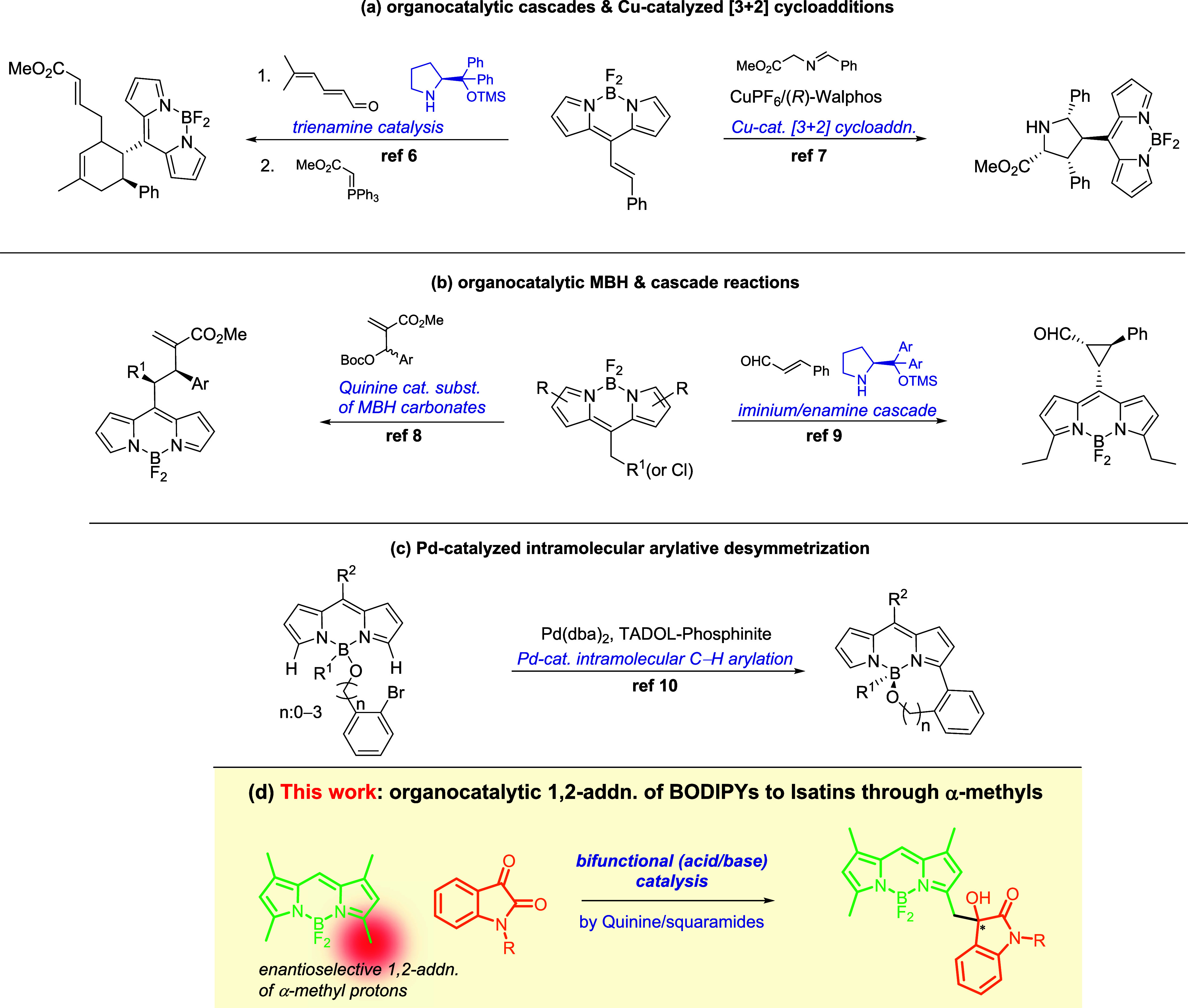
Catalytic enantioselective transformations of achiral BODIPYs.

In recent years, there has been a surge of interest in chiral BODIPYs for their potential in chiroptical applications.^5−10^ However, conventional synthesis of chiral BODIPYs often involves a synthesis of racemic mixtures followed by laborious and expensive chiral separations using *preparative* high-performance liquid chromatography (HPLC), significantly hindering their scalability and accessibility. Catalytic asymmetric synthesis has emerged as a powerful tool to address limitations in accessing enantiomerically enriched BODIPYs.^6−10^ Pioneering work by Alemán and co-workers in 2019 demonstrated the first example of BODIPYs as prochiral substrates for such transformations, achieving the synthesis of chiral cyclohexenyl-BODIPYs via trienamine catalysis with dienals.^6^ Later, they further expanded this methodology to obtain chiral pyrrolidine-incorporated BODIPYs using asymmetric copper-catalyzed [3 + 2] cycloaddition reactions.^7^ Building on this progress, in 2021, Rios and co-workers reported the first enantioselective substitution of Morita–Baylis–Hillman (MBH) carbonates with *meso*-alkyl-BODIPYs, achieved in the presence of cinchona alkaloids (Figure 1).^8^

More recently, Rios and Veselý developed a highly efficient asymmetric organocatalytic cascade reaction involving highly activated *meso*-chloromethylBODIPYs and α,β-unsaturated aldehydes, leading to chiral cyclopropane-bearing BODIPYs with three stereocenters.^9^ In addition to these organocatalytic advancements, Li and He very recently reported a palladium-catalyzed intramolecular C–H arylation reaction enabling the enantioselective synthesis of boron-stereogenic BODIPYs via a desymmetrization strategy.^10^

Chiral squaramide-based organocatalysts, pioneered by Rawal,^11^ have emerged as powerful tools for synthesizing enantiomerically enriched compounds due to their efficient hydrogen-bonding abilities.^12^ Our research group has a longstanding interest in exploring the potential of such catalysts in various organic transformations.^13−18^ We have successfully employed our own bifunctional squaramide catalysts in reactions like asymmetric Michael additions^13^ and Friedel–Crafts alkylation,^14^ achieving excellent results. This approach has also been extended to other reactions including aza-Henry,^15^ Sulfa-Michael addition,^16^ Mannich,^17^ and some domino^18^ reactions.

## Results and Discussion

To further explore the potential of our bifunctional chiral quinine-derived squaramides (**I**–**III**, Table 1), we investigated the possibility of enantioselectively functionalizing the chemically weakly activated α-methyl protons of 1,3,5,7-tetramethyl-BODIPYs (**1a**). We selected 1,3,5,7-tetramethyl-BODIPYs due to (1) their ready accessibility, either through well-established chemical synthesis or commercial availability, and (2) the unprecedented organocatalytic activation of their α-methyl protons in a new stereoselective transformation.

**Table 1:**
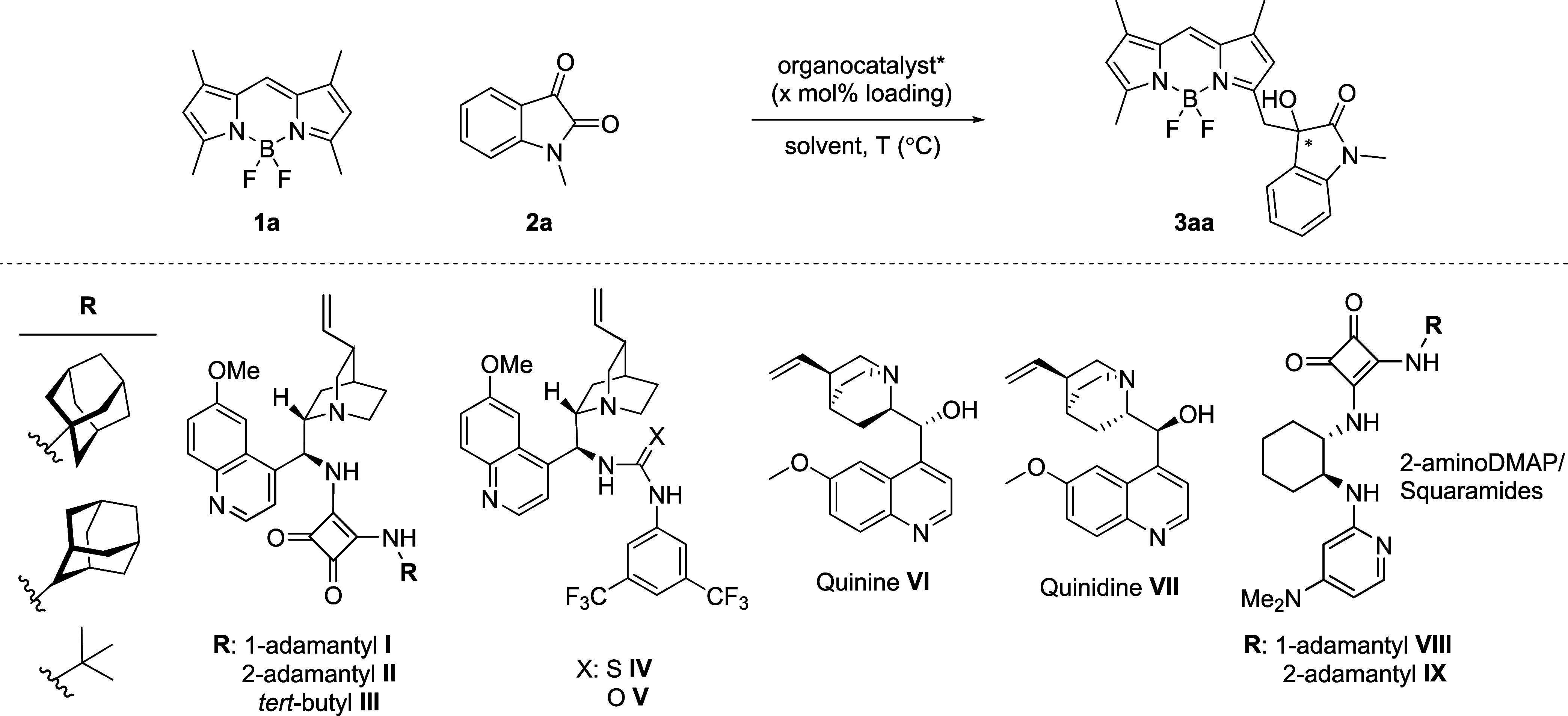
Optimization Studies^a^^,^^b^

entry	catalyst	solvent	time (day)	yield^c^ (%)	ee^d^ (%)
1	**I**	DCM	11	7	17
2	**II**	DCM	6	6	38
3	**III**	DCM	6	8	23
4	**IV**	DCM	10	17	30
5	**V**	DCM	10	40	18
6	**VI**	DCM	6	27	6
7	**VII**	DCM	8	26	4
8	**VIII**	DCM	9	27	3
9	**IX**	DCM	9	44	7
10	**II**	DCM	14	10	51
11	**II**	CHCl_3_	9	10	38
12	**II**	1,2-DCE	7	13	40
13	**II**	PhCH_3_	12	6	31
14	**II**	THF	7	16	25
15	**II**	CH_3_CN	6	9	*rac*
16	**II**	1,4-dioxane	8	10	25
17^e^	**II**	DCM	4	29	30
18^e^	**II**	1,2-DCE	4	29	32
19^f^	**II**	1,2-DCE	4	27	16
20^g^	**II**	1,2-DCE	3	34	11
21^h^	**II**	DCM	11	3	34
22^i^	**II**	DCM	8	6	30
23^j^	**II**	DCM	7	10	53
24^j^^,^^k^	**II**	DCM	6	7	32
25^j^^,^^l^	**II**	DCM	11	5	36
26^j^^,^^m^	**II**	DCM	0.02	22	*rac*
27^j^^,^^n^	**II**	DCM	1	20	15

a Reaction conditions: BODIPY **1a** (0.4 M), isatin **2a** (1 equiv), organocatalysts **I**–**VII** (20 mol %).

b For entries 10–27, isatin **2a** was used in 2 equiv.

c Isolated yields.

d Determined by chiral HPLC.

e Reaction conducted at 40 °C.

f Reaction conducted at 60 °C.

g Reaction conducted at 80 °C.

h [**1a**] = 0.1 M.

i [**1a**] = 0.2 M.

j [**1a**] = 0.5 M.

k 30 mol % organocatalyst.

l 10 mol % organocatalyst.

m 100 mol % DBU was used as external base.

n 100 mol % Cs_2_CO_3_ was used as external base.

Isatins were chosen as promising reaction partners due to their frequent use in catalytic asymmetric reactions and the diverse biological activities associated with the corresponding chiral 3-hydroxy-3-substituted 2-oxindole adducts.^19^

We commenced our catalysis works by reacting an equimolar mixture of BODIPY **1a** and isatin **2a** in dichloromethane (DCM) with a catalyst loading of 20 mol % at room temperature (Table 1, entries 1–9). DCM was chosen for initial catalyst screening studies due to its balanced polarity promoting optimal catalyst–substrate interactions for H-bonding activation. Among several screened bifunctional chiral organocatalysts (quinine/squaramides **I**–**III**, quinine/(thio)ureas **IV**–**V**,^20^ quinine **VI**, quinidine **VII**, and 2-aminoDMAP/squaramides^13a^**VIII** and **IX**), catalyst **II** provided the most promising results, giving the desired product **3aa** with the 38% enantiomeric excess (*ee*) but a modest yield of 6% (Table 1, entry 2). Doubling the equivalency of isatin **2a** (**2a**:**1a** = 2:1) significantly improved the enantioselectivity to 51% *ee*, with a corresponding increase in yield to 10% (Table 1, entry 10). Exploration of alternative solvents like chloroform, 1,2-dichloroethane, toluene, THF, acetonitrile, and 1,4-dioxane failed to yield any improvements in either enantioselectivity or yield (Table 1, entries 11–16). As the reactions were gradually heated in either DCM or 1,2-DCE, a corresponding decline in enantioselectivity was observed (entries 17–20). We then examined the effect of BODIPY **1a** concentration on the reaction (Table 1, entries 21–23). After screening concentrations of 0.1, 0.2, and 0.5 M, we identified 0.5 M as optimal and used this concentration for subsequent catalyst loading optimization. Unfortunately, deviating from the 20 mol % catalyst loading proved detrimental, as both increasing it to 30 mol % and decreasing it to 10 mol % resulted in diminished enantioselectivity (Table 1, entries 24 and 25). To further enhance the reaction rate, in addition to catalyst **II**, the use of external bases such as 1,8-diazabicyclo[5.4.0]undec-7-ene (DBU) or cesium carbonate led to faster transformations, but with considerably reduced selectivity (entries 26 and 27). Eventually, under optimized reaction conditions (20 mol % catalyst **II**, 0.5 M BODIPY **1a** in DCM, 2 equiv isatin **2a** at room temperature; Table 1, entry 23), this protocol afforded access to nine novel chiral BODIPY-oxindole dyes (**3**) employing various BODIPYs (**1**) and isatins (**2**) (see Table 2).

**Table 2:**
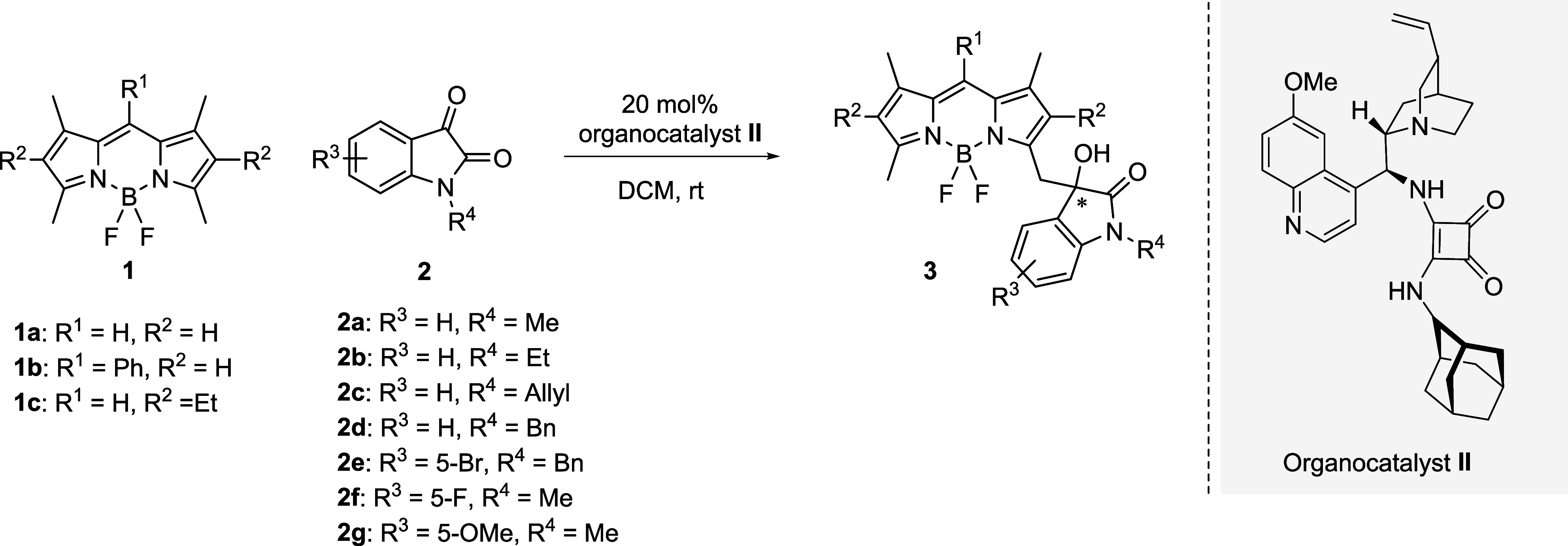

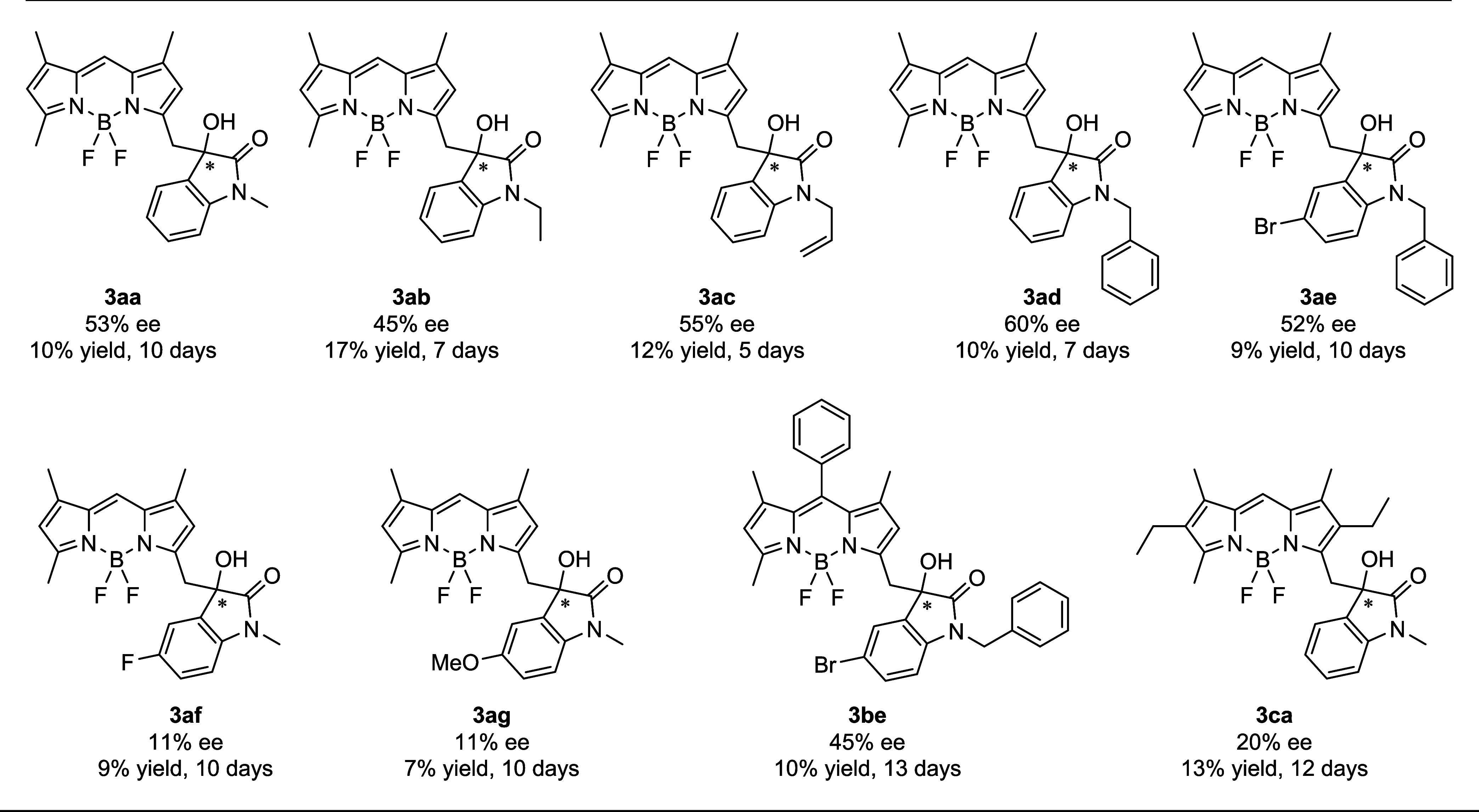
Derivatization Study^a^^,^^b^

a Isolated yields.

b ee values are determined on a chiral HPLC system.

Reactions typically require 1–2 weeks of stirring to achieve reasonable conversions. We initially further explored *N*-substituted isatins (**2b**–**2d**) and observed the highest enantioselectivity (60%) with compound **2d**, affording product **3ad**. To investigate the effect of substituents on the isatin’s aromatic ring, we included 5-bromo-substituted *N*-benzyl (**2e**), 5-fluoro- (**2f**), and 5-methoxy-substituted *N*-methyl (**2g**) isatins in the reaction scheme. While the reactions’ efficiency appeared to be insensitive to the electronic nature of the isatins (**2f** vs **2g**), giving similar yields over the same reaction periods, the deterioration of enantioselectivity in **2f** and **2g** compared to **2a** or **2e** was perplexing. To diversify the reaction on BODIPY side, we further evaluated a *meso*-phenyl derivative (**1b**) and a 2,6-diethyl substituted derivative (**1c**) of **1a**, both of which were successfully introduced into the reaction. The 2,6-diethyl-substituted derivative (**1c**) was selected for its anticipated absorption and emission at longer wavelengths (orange–red) to demonstrate the tunability of the emission wavelength. To further expand the scope of acceptors, we also investigated the reaction of 1,3,5,7-tetramethyl-BODIPY with alternative electrophiles, including *trans*-β-nitrostyrene, 4-nitrobenzaldehyde, and butane-2,3-dione. Unfortunately, these reactions did not yield any product, as confirmed by thin layer chromatography (TLC) and nuclear magnetic resonance (NMR) analysis. Despite achieving modest enantioselectivities and yields under extended reaction times and high catalyst loadings (20 mol %), this work still represents a significant advancement in catalytic asymmetric synthesis of chiral BODIPYs. Our platform offers the first example of organocatalytic activation of the *low-acidity α*-methyl protons on a common, readily available 1,3,5,7-tetramethyl-BODIPY skeleton.

**Figure 2:**
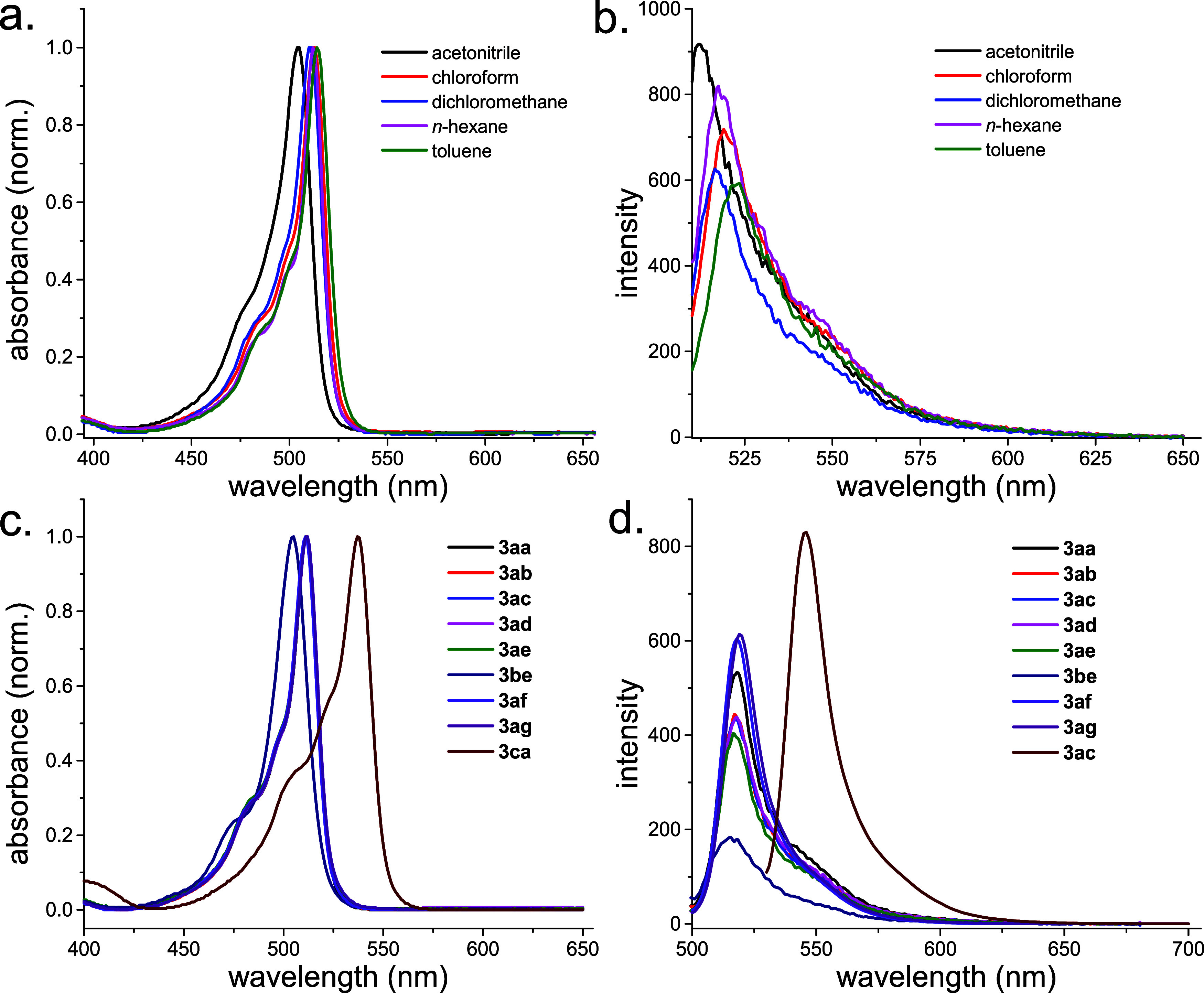
Solvent effect on (a) UV–vis absorption and (b) fluorescence emission spectra of **3aa** (2 μM). **(c)** UV–vis absorption (left) and (d) fluorescence emission spectra of BODIPY derivatives **3** (1 μM in CHCl_3_). Excitation and emission slits were set to 5.0 nm for all fluorescence measurements.

Due to the electronically decoupled nature of the BODIPY and oxindole units, separated by a methylene spacer, we investigated the influence of solvent on the ultra-violet visible (UV-vis) absorbance and fluorescence behavior of product **3aa**. As expected for such a system, **3aa** exhibited minimal solvent dependency across all tested solvents. Only slight bathochromic shifts were observed in both absorption and emission spectra (Figure 2a,b).

This behavior suggests weak interaction between the BODIPY fluorophore and the surrounding solvent molecules, similar to its parent BODIPYs. Additionally, fluorescence analysis revealed high emission intensity with excitation and emission slits set at 3.5 nm. This indicates that the peripheral chemical modification does not significantly affect the emission properties of the BODIPY fluorophore. All derivatives which carry 1,3,5,7-tetramethyl-substituents on BODIPY chromophore displayed a central absorption band around 510 nm. Substitutions on the isatin core had negligible effects on the absorption maxima, as seen in Figure 2c. While the *meso*-phenyl substituted BODIPY derivative (**3be**) exhibited a slightly blue-shifted absorption maximum at 506 nm, the 2,6-diethyl substituted derivative **1c** showed an approximately 30 nm red shift, with absorption peaking at 538 nm. These examples clearly demonstrate the color tunability potential of this system. Notably, all derivatives except **3be** exhibited high fluorescence intensity, indicating a relatively lower response for the *meso*-phenyl substituted compound. This lower emission in **3be** could be attributed to the introduction of the phenyl group at the *meso*-position, potentially hindering structural rigidity and reducing excited-state stability. All of the BODIPY-oxindole dyes (**3**) exhibited small Stokes shifts (8–12 nm) characteristic of the parent fluorophore, as well. Given the high emission observed for most derivatives, we determined the fluorescence quantum yield of the representative product, **3aa** using fluorescein as the standard.^21,22^ We calculated a quantum yield of 0.78 for **3aa**, comparable also to its parent BODIPY.^1^

**Figure 3:**
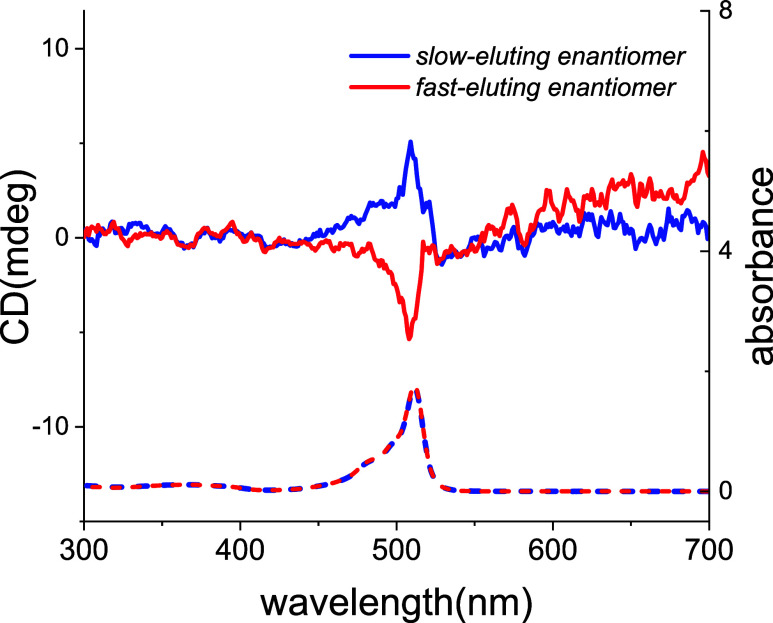
ECD spectra of separated enantiomers in chloroform (20 μM).

Enantioenriched product **3aa** was analyzed using electronic circular dichroism (ECD) spectroscopy in CHCl_3_ solutions at varying concentrations. However, consistently weak ECD signals were observed. This can be attributed to two factors: (1) the physical separation between the chromophore and the stereocenter in the molecule, and (2) the presence of both enantiomers in solution (76.5:23.5 ratio) leading to partial cancellation of the ECD signal due to their inherent mirror-image relationship. To address this limitation and demonstrate the ECD activity of these novel dyes, the racemic form of **3aa** was successfully separated into its individual enantiomers using preparative HPLC (see Supporting Information (SI) file for chromatograms). Subsequent ECD spectra recorded for the isolated enantiomers in CHCl_3_ solutions (each at 20 μM concentration) displayed observable Cotton effects peaking at 511 nm in the visible region, coinciding with the UV–vis absorption maximum. This observation signifies the chiral character of the adducts (Figure 3). Notably, the ECD spectra of the separated enantiomers mirrored the UV–vis spectra, exhibiting similar shapes and characteristic mirror image symmetry across the electromagnetic radiation range studied.

We also sought to understand the sense of chirality induction with catalyst **II**. To this end, we aimed to determine the absolute stereochemical configuration of bromine-containing derivative **3ae** through anomalous X-ray scattering. However, despite multiple attempts, we were unable to obtain single crystals suitable for this analysis. Crystallization by slow evaporation in various solvent systems, including *n*-hexane:DCM binary mixtures, methanol, and acetone, was unsuccessful. Consequently, to assign the absolute stereochemical configuration of the model product **3aa**, we performed density functional theory (DFT) calculations using Gaussian 16, Revision C.02 at the B3LYP/6-311G(d) theoretical level with DFT-D3 dispersion correction followed by conformational analysis by classical methods (Figure S1).^23−27^ Thirty singlet excited states were calculated for the lowest energy geometry by TDDFT in chloroform as an implicit solvent using the integral equation formalism PCM.^28^ Natural transition orbitals (NTOs) were calculated to explain the observed transitions.^29^ Additional calculations including organocatalysts were conducted in DCM to mimic experimental conditions and to understand the effect of reactant conformations on the final structure.

**Figure 4:**
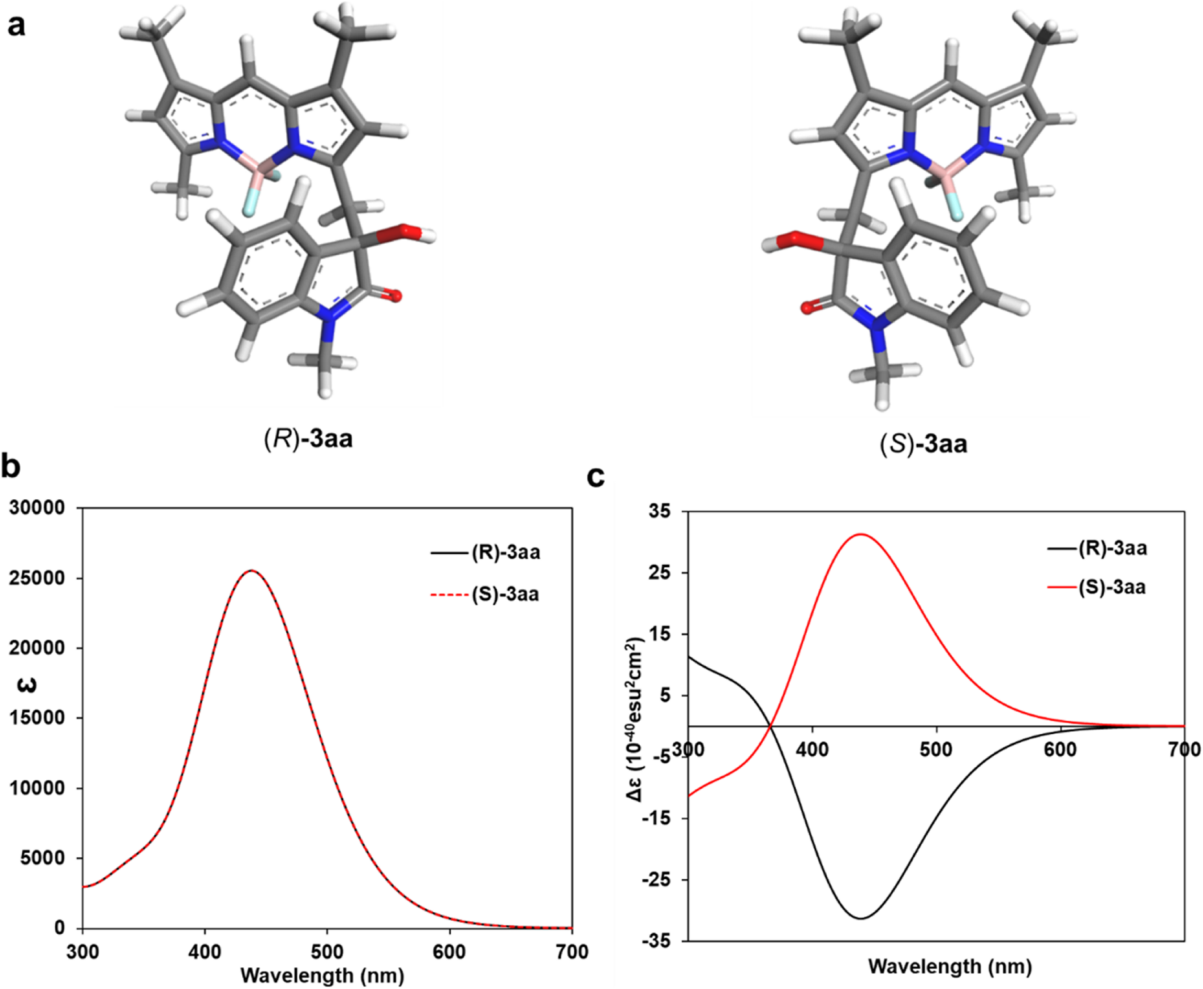
Calculated (a) geometry-optimized structures: (*R*)-**3aa** vs (*S*)-**3aa**, (b) UV–vis spectra, and (c) ECD spectra for these structures.

The calculated *R* and *S* enantiomers for **3aa** are shown in Figure 4a. The computed UV–vis spectra, depicted in Figure 4b, exhibit perfectly identical spectral features but with about 60 nm blue-shifted absorption maxima compared to the experimental results. The ECD spectra calculated for these structures display a perfect mirror-like relationship (Figure 4c), with two distinct peaks for the *R* and *S* enantiomers centered around 450 nm, closely matching the experimental observations.

To gain insight into the electronic transitions, natural transition orbitals (NTO, vide infra) for the key singlet excitations with highest oscillatory frequencies were calculated (Figure S2). Figure S2 shows that the excitation possesses corresponding to the experimental spectra exhibits a charge-transfer (CT) transition character.

**Figure 5:**
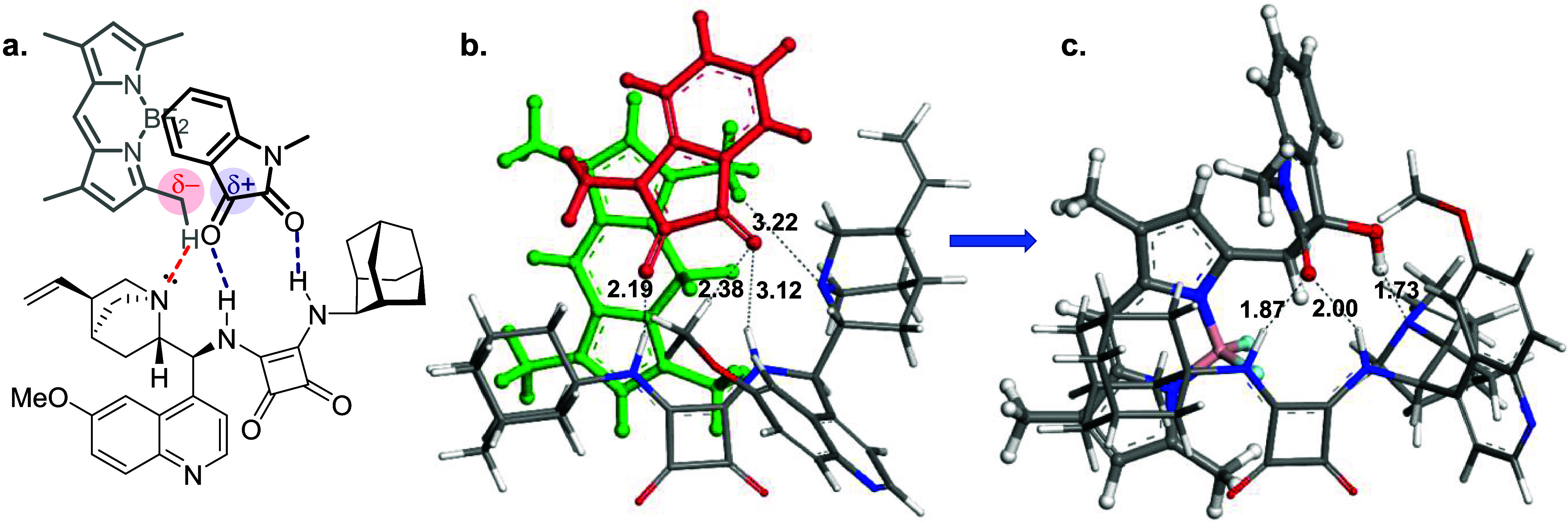
(a) Putative pretransition state (pre-TS) model for **3aa**. (b) Lowest energy reactant interactions in the presence of organocatalyst and (c) the potential (*R*)**-3aa** product formed from this conformation of the reactants **1a** and **2a**.

The lowest-energy structures of the model substrates **1a** and **2a** were determined in the presence of the quinine/squaramide-based organocatalyst (**II**), starting from various initial conformations. The lowest-energy structures for the reactants and the resulting product from this conformation and configuration are shown in Figure 5. Our calculations indicate that the *R* enantiomer is favored by these lowest-energy interactions and is predicted to be the dominant product (Figure 5c). The proposed pretransition state (pre-TS) model (Figure 5a) illustrates the key interactions between the catalyst (**II**) and the model substrates (**1a** and **2a**).

This model depicts the spatial arrangement prior to bond formation and highlights the bifunctional activation of both the BODIPY and the isatin substrates. Comparing the performance of quinine-based catalysts in the model reaction (Table 1), particularly quinine (**VI**) and quinidine (**VII**) with quinine-squaramides and -(thio)-ureas (**I**–**V**), we conclude that the reaction is primarily promoted by the basic domain of the catalysts, specifically the quinuclidine moiety. This is evident from the comparable and superior yields observed with quinine (**VI**) and quinidine (**VII**) despite their lack of double H-bonding capacity seen in squaramides or (thio)ureas (**I**–**V**). The dependence of reaction kinetics on catalyst basicity is further supported by the high yields obtained from the *superbasic* 2-aminoDMAP-incorporating catalysts (**VIII** and **IX**). However, the diminished enantioselectivities observed with these catalysts remain unclear.

The quinine (**VI**)/quinidine (**VII**) pair distinctly differs from the remaining catalysts by possessing only a single hydrogen bond donor (−OH), which may explain the lower enantioselectivities observed with these catalysts compared to the more bifunctionally active double H-bond donors (**I**–**V**). The double hydrogen-bond donating catalysts (**I**–**V**) likely rigidify the transition state, restricting the rotations of the isatin during preorganization in the chiral pocket, which may account for their relatively higher stereoselectivity. Quantum chemical calculations indicate that the interaction between the α-methyl group of the BODIPYs and the catalysts apparently involves only a single hydrogen bond through coordination with the basic nitrogen of the organocatalysts. Interestingly, the calculations also suggest that the methoxy hydrogen of the quinoline unit in catalyst **II** contributes to the activation of the isatin substrate alongside the double H-bonding interaction of the squaramide unit.

## Conclusions

In conclusion, this work presents the first instance of an enantioselective addition reaction between readily available 1,3,5,7-tetramethyl-BODIPYs (**1**) and *N*-substituted isatins (**2**). We achieved this advancement using a bifunctional 2-adamantyl-containing squaramide/quinine organocatalyst (**II**) at a moderate loading of 20 mol %. This approach provides access to a library of nine novel BODIPY-oxindole tertiary alcohols (**3aa**–**3ca**) with promising fluorescence properties. While enantioselectivities remain modest (up to 60% *ee*), all derivatives exhibit high emission intensity with tunable emission colors. Notably, a representative example (**3aa**) displayed a very high quantum yield (0.78) comparable to fluorescein. Furthermore, successful chiral HPLC separation of enantiomers and the Cotton effects observed in their ECD spectra confirm the chirality of these BODIPY dyes. Quantum chemical calculations helped assign the absolute stereochemical configuration of the model oxindole **3aa** as (*R*) and provided insight into the catalyst–substrate key interactions.

## Experimental Section

### General Information

The ^1^H and ^13^C NMR spectra of compounds were recorded using a Bruker Spectroscopin Avance DPX 400 spectrometer with CDCl_3_ as the solvent. Spin multiplicities were denoted as follows: bs (broad singlet), s (singlet), d (doublet), dd (doublet of doublet), t (triplet), and m (multiplet). Chemical shift values were reported in parts per million (ppm) using TMS as the reference, and the coupling constants of the compounds were expressed in Hertz (Hz). HPLC analyses were performed using an Agilent HPLC system equipped with Daicel Chiralpak OD-H, AD-H, AS-H, and OJ-H columns, operated at room temperature. For chirality assay determinations, isocratic systems comprising mixtures of HPLC-grade *n*-hexane and isopropyl alcohol were used as mobile phases. Preparative separations of enantiomers were carried out using an Agilent Technologies Preparative HPLC-1200 Series system with a diode array detector (DAD) and a Kromasil 10-Cellucoat column (1.0 cm × 25 cm) at UNAM, Bilkent University. Optical rotations were measured using a Rudolph Scientific Autopol III polarimeter, and specific rotations were reported as [α]_D_^25^ (*c* in g/100 mL, solvent). Additionally, HRMS data were obtained using an Agilent 6224 TOF LC-MS at Unam, Bilkent University. Functional group determinations were carried out via Fourier transform infrared spectroscopy (FTIR) analysis using a Bruker α Platinum ATR, with band positions reported in cm^–1^. The melting points of the products were determined using a MEL-TEMP 1002D apparatus. For spectroscopic analyses, the absorption spectra of the compounds were recorded using a Shimadzu UV-2450 UV–vis spectrophotometer, while their emission spectra were analyzed with a PerkinElmer LS55 fluorescence spectrometer. All absorption and emission analyses were conducted using a quartz cell with a 1 cm path length. The chiroptical properties of the products were examined through ECD spectra obtained using a JASCO J-1500 CD spectrometer. All reactions were monitored using Merck Silica Gel 60 F254 precoated silica gel TLC plates, visualized under UV and visible light. Flash column chromatography was performed using Silica Gel 60 F254 with mesh sizes of 200–400 and 60–200. Bifunctional organocatalysts **I**–**V**^13b,20^ and **VIII**–**IX**^13a^ were synthesized following literature procedures.

### General Experimental Procedures

#### Procedure for Racemic Syntheses

DBU (20 mol %) was added to a solution of BODIPY derivative **1** (0.1 mmol) and isatin derivative **2** (0.2 mmol) in dichloromethane (DCM, 0.2 mL). The reaction mixture was stirred at room temperature for 5–15 min. After evaporating the DCM under reduced pressure, the crude product was directly subjected to flash column chromatography using *n*-hexane:ethyl acetate eluant systems (1:5 to 1:2) for purification.

#### Optimized Procedure for Organocatalytic Asymmetric Syntheses

A mixture of BODIPY derivative **1** (0.1 mmol), isatin derivative **2** (0.2 mmol), and bifunctional organocatalyst **II** (0.02 mmol) was stirred in DCM (0.2 mL) at room temperature for 5–14 days. All reactions were monitored by TLC and then, purified with column chromatography using *n*-hexane:ethyl acetate eluant systems (1:5 to 1:2) for purification.

##### Data for BODIPY (*R*)-**3aa**

Following the general procedure, chiral BODIPY **3aa** was obtained as an orange solid with an isolated yield of 10% after 7 days. Melting Point: 164–168 °C. HPLC analysis (AD-H, *n*-Hexane/Isopropyl alcohol, 80:20, 1.0 mL/min, 254 nm) *t*_major_ = 15 min and *t*_minor_ = 23 min, 53% ee, [α]_D_^25^= −105.8 (*c* 0.1, CH_2_Cl_2_). ^1^H NMR (400 MHz, Chloroform-*d*) δ 7.34–7.26 (m, 2H), 7.09 (s, 1H), 7.03 (t, *J* = 7.5 Hz, 1H), 6.83 (d, *J* = 7.7 Hz, 1H), 6.25 (s, 1H), 6.07 (s, 1H), 3.86 (bs, 1H), 3.59 (d, *J* = 15.0 Hz, 1H), 3.31 (d, *J* = 15.0 Hz, 1H), 3.21 (s, 3H), 2.51 (s, 3H), 2.29 (s, 3H), 2.26 (s, 3H). ^13^C NMR (101 MHz, CDCl_3_) δ 177.1, 158.6, 151.9, 143.1, 142.6, 140.1, 134.0, 132.8, 129.9, 129.6, 124.5, 122.7, 120.6, 119.7, 119.5, 108.2, 75.2, 36.6, 26.2, 14.8, 11.3, 11.12 ppm. IR (neat): 3380, 2917, 1699, 1600, 1507, 1469, 1376, 1351, 1314, 1247, 1193, 1150, 1065, 1029, 964, 898, 829, 751, 702, 671, 579, 537, 479, 423 cm^–1^. HRMS (ESI-TOF) *m*/*z*: [M]^+^ Calcd for C_22_H_22_BF_2_N_3_O_2_ 409.1773; Found 409.1776.

##### Data for BODIPY **3ab**

Following the general procedure, chiral BODIPY **3ab** was obtained as an orange solid with an isolated yield of 17% after 7 days. Melting Point: 184–186 °C (decomp.). HPLC analysis (AD-H, *n*-Hexane/Isopropyl alcohol, 80/20, 1.0 mL/min, 510 nm) *t*_major_ = 15 min and *t*_minor_ = 22 min, 45% ee, [α]_D_^25^ = −99.0 (*c* 0.1, CH_2_Cl_2_). ^1^H NMR (400 MHz, Chloroform-*d*) δ 7.33–7.27 (m, 2H), 7.09 (s, 1H), 7.03 (t, *J* = 7.5 Hz, 1H), 6.85 (d, *J* = 7.7 Hz, 1H), 6.22 (s, 1H), 6.07 (s, 1H), 3.86–3.65 (m, 3H), 3.59 (d, *J* = 15.1, 1H), 3.32 (d, *J* = 15.1 Hz, 1H), 2.51 (s, 3H), 2.27 (d, *J* = 8.8 Hz, 6H), 1.28 (t, *J* = 7.2 Hz, 3H). ^13^C NMR (101 MHz, CDCl_3_) δ 176.6, 158.6, 151.8, 142.6, 142.2, 140.1, 134.0, 132.8, 130.1, 129.5, 124.7, 122.5, 120.6, 119.7, 119.5, 108.4, 75.1, 36.7, 34.7, 14.8, 12.4, 11.3, 11.2 ppm. IR (neat): 3382, 2919, 1712, 1602, 1512, 1470, 1431, 1367, 1312, 1241, 1211, 1174, 1152, 1133, 1076, 1032, 966, 898, 878, 819, 753, 700, 673, 613, 575, 498, 473 cm^–1^. HRMS (ESI-TOF) *m*/*z*: [M – H]^−^ Calcd for C_23_H_23_BF_2_N_3_O_2_ 422.1851; Found 422.1881.

##### Data for BODIPY **3ac**

Following the general procedure, chiral BODIPY **3ac** was obtained as an orange solid with an isolated yield of 12% after 5 days. Melting Point: 212–214 °C (decomp.). HPLC analysis (AD-H, *n*-Hexane/Isopropyl alcohol, 80/20, 1.0 mL/min, 254 nm) *t*_major_ = 15 min and *t*_minor_ = 22 min, 55% ee, [α]_D_^25^ = −161.5 (*c* 0.1, CH_2_Cl_2_). ^1^H NMR (400 MHz, Chloroform-*d*) δ 7.34–7.27 (m, 2H), 7.09 (s, 1H), 7.03 (t, *J* = 7.4 Hz, 1H), 6.83 (d, *J* = 7.7 Hz, 1H), 6.22 (s, 1H), 6.08 (s, 1H), 5.91–5.79 (m, 1H), 5.24 (t, *J* = 14.1 Hz, 2H), 4.44 (d, *J* = 16.3 Hz, 1H), 4.23 (d, *J* = 16.2 Hz, 1H), 3.80 (bs, 1H), 3.62 (d, *J* = 15.0 Hz, 1H), 3.35 (d, *J* = 15.0 Hz, 1H), 2.52 (s, 3H), 2.27 (d, *J* = 8.0 Hz, 6H). ^13^C NMR (101 MHz, CDCl_3_) δ 176.8, 158.7, 151.7, 142.6, 142.3, 140.1, 134.1, 132.8, 131.2, 129.9, 129.5, 124.6, 122.7, 120.6, 119.7, 119.5, 117.7, 109.1, 75.1, 42.4, 36.8, 14.8, 11.3, 11.2 ppm. IR (neat): 3399, 2960, 2918, 2851, 1714, 1601, 1511, 1469, 1428, 1357, 1250, 1199, 1174, 1150, 1076, 973, 931, 896, 875, 817, 799, 756, 703, 672, 610, 580, 558, 472 cm^–1^. HRMS (ESI-TOF) *m*/*z*: [M + Na]^+^ Calcd for C_24_H_24_BF_2_N_3_NaO_2_ 458.1827; Found 458.1837.

##### Data for BODIPY **3ad**

Following the general procedure, chiral BODIPY **3ad** was obtained as an orange solid with an isolated yield of 10% after 7 days. Melting Point: 211–213 °C (decomp.). HPLC analysis (AS-H, *n*-Hexane/Isopropyl alcohol, 75/25, 1.0 mL/min, 510 nm) *t*_minor_ = 22 min and *t*_major_ = 33 min, 60% ee, [α]_D_^25^ = −127.4 (*c* 0.1, CH_2_Cl_2_). ^1^H NMR (400 MHz, Chloroform-*d*) δ 7.37–7.23 (m, 6H), 7.19 (t, *J* = 7.8 Hz, 1H), 7.09 (s, 1H), 7.01 (t, *J* = 7.5 Hz, 1H), 6.71 (d, *J* = 7.8 Hz, 1H), 6.16 (s, 1H), 6.08 (s, 1H), 5.01 (d, *J* = 15.7 Hz, 1H), 4.79 (d, *J* = 15.7 Hz, 1H), 3.90 (bs, 1H), 3.69 (d, *J* = 15.0 Hz, 1H), 3.41 (d, *J* = 14.9 Hz, 1H), 2.53 (s, 3H), 2.26 (s, 6H). ^13^C NMR (101 MHz, CDCl_3_) δ 177.3, 158.8, 151.5, 142.7, 142.3, 140.1, 135.5, 134.1, 132.8, 129.9, 129.5, 128.7, 127.5, 127.3, 124.6, 122.8, 120.6, 119.8, 119.4, 109.3, 75.2, 43.8, 36.8, 14.8, 11.3, 11.2 ppm. IR (neat): 3354, 2960, 2921, 2852, 1691, 1599, 1583, 1529, 1511, 1494, 1469, 1444, 1408, 1381, 1302, 1254, 1235, 1194, 1169, 1149, 1068, 972, 903, 795, 746, 727, 707, 691, 672, 622, 582, 546, 479, 456 cm^–1^. HRMS (ESI-TOF) *m*/*z*: [M + Na]^+^ Calcd for C_28_H_26_BF_2_N_3_NaO_2_ 508.1984; Found 508.1984.

##### Data for BODIPY **3ae**

Following the general procedure, chiral BODIPY **3ae** was obtained as an orange solid with an isolated yield of 9% after 10 days. Melting Point: 190–195 °C (decomp.). HPLC analysis (OD-H, *n*-Hexane/Isopropanol, 90/10, 1.0 mL/min, 510 nm) *t*_minor_ = 22 min and *t*_major_ = 46 min, 52% ee, [α]_D_^25^ = −109.2 (*c* 0.1, CH_2_Cl_2_). ^1^H NMR (400 MHz, Chloroform-*d*) δ 7.42 (d, *J* = 1.7 Hz, 1H), 7.36–7.27 (m, 6H), 7.12 (s, 1H), 6.57 (d, *J* = 8.3 Hz, 1H), 6.11 (d, *J* = 2.6 Hz, 2H), 4.98 (d, *J* = 15.7 Hz, 1H), 4.78 (d, *J* = 15.7 Hz, 1H), 4.11 (bs, 1H), 3.68 (d, *J* = 14.9 Hz, 1H), 3.32 (d, *J* = 14.9 Hz, 1H), 2.55 (s, 3H), 2.28 (s, 6H). ^13^C NMR (101 MHz, CDCl_3_) δ 176.8, 159.4, 150.3, 143.2, 141.2, 140.0, 135.0, 134.3, 132.8, 132.3, 131.9, 128.8, 128.1, 127.7, 127.2, 120.8, 120.1, 119.4, 115.4, 110.8, 75.2, 43.9, 36.7, 14.8, 11.3, 11.3 ppm. IR (neat): 3258, 2920, 2851, 1689, 1603, 1531, 1507, 1483, 1450, 1431, 1410, 1370, 1342, 1307, 1293, 1241, 1216, 1192, 1170, 1153, 1122, 1078, 1016, 973, 920, 897, 871, 806, 758, 699, 627, 606, 557, 533, 468 cm^–1^. HRMS (ESI-TOF) *m*/*z*: [M + Na]^+^ Calcd for C_28_H_25_BBrF_2_N_3_NaO_2_ 586.1089; Found 586.1088.

##### Data for BODIPY **3af**

Following the general procedure, chiral BODIPY **3af** was obtained as an orange solid with an isolated yield of 9% after 10 days. Melting Point: 186–188 °C (decomp.). HPLC analysis (AD-H, *n*-Hexane/Isopropyl alcohol, 80:20, 1.0 mL/min, 510 nm) t_major_ = 13 min and *t*_minor_ = 26 min, 11% ee, [α]_D_^25^ = −10.0 (*c* 0.1, CH_2_Cl_2_). ^1^H NMR (400 MHz, Chloroform-*d*) δ 7.11 (s, 1H), 7.00 (ddd, *J* = 9.0, 5.1, 2.6 Hz, 2H), 6.75 (dd, *J* = 9.0, 4.2 Hz, 1H), 6.25 (s, 1H), 6.09 (s, 1H), 3.93 (s, 1H), 3.59 (d, *J* = 15.1 Hz, 1H), 3.27 (d, *J* = 15.2 Hz, 1H), 3.20 (s, 3H), 2.51 (s, 3H), 2.28 (d, *J* = 13.0 Hz, 6H). ^13^C NMR (101 MHz, CDCl_3_) δ 177.0, 159.1, 159.1 (d, *J* = 241 Hz), 151.0, 143.0, 140.0, 139.0, 134.2, 132.8, 131.4 (d, *J* = 8.1 Hz), 120.7, 119.9, 119.4, 115.7 (d, *J* = 23.6 Hz), 112.9 (d, *J* = 25.2 Hz), 108.7 (d, *J* = 8.1 Hz), 75.3, 36.5, 26.3, 14.8, 11.3, 11.2 ppm. IR (neat): 3371, 3101, 3047, 2966, 2922, 1715, 1601, 1512, 1471, 1420, 1357, 1308, 1269, 1248, 1228, 1194, 1168, 1142, 1124, 1078, 1030, 970, 921, 900, 871, 828, 811, 793, 755, 724, 710, 700, 673, 633, 598, 576, 555, 471, 461, 423 cm^–1^. HRMS (ESI-TOF) *m*/*z*: [M – H]^+^ Calcd for C_22_H_21_BF_3_N_3_O_2_ 426.1601; Found 426.1636.

##### Data for BODIPY **3ag**

Following the general procedure, chiral BODIPY **3ag** was obtained as an orange solid with an isolated yield of 7% after 10 days. Melting Point: 135–140 °C (decomp.). HPLC analysis (AD-H, *n*-Hexane/Isopropyl alcohol, 80:20, 1.0 mL/min, 510 nm) *t*_major_ = 19 min and *t*_minor_ = 27 min, 11% ee, [α]_D_^25^ = −58.0 (*c* 0.1, CH_2_Cl_2_). ^1^H NMR (400 MHz, Chloroform-*d*) δ 7.08 (s, 1H), 6.92 (d, *J* = 2.6 Hz, 1H), 6.83 (dd, *J* = 8.5, 2.6 Hz, 1H), 6.73 (d, *J* = 8.5 Hz, 1H), 6.23 (s, 1H), 6.07 (s, 1H), 3.73 (s, 3H), 3.61 (d, *J* = 15.0 Hz, 1H), 3.30 (d, *J* = 15.0 Hz, 1H), 3.18 (s, 3H), 2.51 (s, 3H), 2.26 (d, *J* = 7.1 Hz, 6H). ^13^C NMR (101 MHz, CDCl_3_) δ 176.9, 158.7, 155.9, 151.8, 142.6, 140.0, 136.5, 134.1, 132.8, 130.9, 120.6, 119.7, 119.5, 114.6, 111.5, 108.7, 75.6, 55.7, 36.7, 26.3, 14.7, 11.3, 11.2 ppm. IR (neat): 3310, 2920, 1689, 1601, 1533, 1496, 1468, 1431, 1365, 1285, 1227, 1152, 1076, 1031, 972, 909, 867, 801, 725, 672, 636, 579, 552, 477 cm^–1^. HRMS (ESI-TOF) *m*/*z*: [M – H]^+^ Calcd for C_23_H_24_BF_2_N_3_O_3_ 438.1801; Found 438.1841.

##### Data for BODIPY **3be**

Following the general procedure, chiral BODIPY **3be** was obtained as an orange solid with an isolated yield of 10% after 13 days. Melting Point: 111–114 °C (decomp.). HPLC analysis (AD-H, *n*-Hexane/Isopropyl alcohol, 90/10, 1.0 mL/min, 254 nm) *t*_major_ = 23 min and *t*_minor_ = 40 min, 45% ee, [α]_D_^25^ = −42 (*c* 0.1, CH_2_Cl_2_). ^1^H NMR (400 MHz, Chloroform-*d*) δ 7.55–7.48 (m, 3H), 7.43 (d, *J* = 1.9 Hz, 1H), 7.36–7.26 (m, 8H), 6.57 (d, *J* = 8.3 Hz, 1H), 6.05 (d, *J* = 11.6 Hz, 2H), 5.01 (d, *J* = 15.7 Hz, 1H), 4.78 (d, *J* = 15.7 Hz, 1H), 3.73 (d, *J* = 15.0 Hz, 1H), 3.36 (d, *J* = 15.0 Hz, 1H), 2.57 (s, 3H), 1.40 (s, 6H), the proton belonging to the OH group was not located. ^13^C NMR (101 MHz, CDCl_3_) δ 176.9, 157.9, 149.1, 145.1, 142.5, 142.0, 141.2, 135.0, 134.6, 132.2, 132.0, 129.2, 129.1, 129.0, 128.9, 128.8, 128.1, 127.7, 127.7, 127.2, 122.1, 121.7, 115.4, 110.8, 75.2, 43.9, 36.6, 14.8, 14.4, 14.3 ppm. IR (neat): 3506, 3379, 2956, 2925, 2856, 1724, 1707, 1609, 1541, 1505, 1481, 1454, 1431, 1407, 1364, 1312, 1260, 1184, 1154, 1077, 1043, 1025, 980, 883, 839, 809, 722, 697, 627, 603, 560, 532, 506, 488, 473 cm^–1^. HRMS (ESI-TOF) *m*/*z*: [M + Na]^+^ Calcd for C_34_H_29_BBrF_2_N_3_NaO_2_ 662.1402; Found 662.1445.

##### Data for BODIPY **3ca**

Following the general procedure, chiral BODIPY **3ca** was obtained as a dark pink solid with an isolated yield of 13% after 12 days. Melting Point: 98–102 °C. HPLC analysis (AD-H, *n*-Hexane/Isopropyl alcohol, 80:20, 1.0 mL/min, 510 nm) *t*_minor_ = 8 min and *t*_major_ = 10 min, 20% ee, [α]_D_^25^ = −46.2 (*c* 0.1, CH_2_Cl_2_). ^1^H NMR (400 MHz, Chloroform-*d*) δ 7.31–7.28 (m, 1H), 7.05 (d, *J* = 4.9 Hz, 2H), 6.97 (q, *J* = 7.5, 6.7 Hz, 1H), 6.82 (d, *J* = 7.8 Hz, 1H), 4.93 (d, *J* = 22.9 Hz, 1H), 3.60 (d, *J* = 15.0 Hz, 1H), 3.21 (s, 4H), 2.52 (s, 3H), 2.40 (q, *J* = 7.6 Hz, 2H), 2.20 (s, 6H), 2.08 (dd, *J* = 14.8, 7.5 Hz, 1H), 1.82 (dd, *J* = 14.8, 7.5 Hz, 1H), 1.07 (t, *J* = 7.6 Hz, 3H), 0.85 (t, *J* = 7.6 Hz, 3H). ^13^C NMR (101 MHz, CDCl_3_) δ 177.5, 157.8, 148.7, 143.0, 138.4, 136.7, 133.5, 133.1, 133.0, 132.8, 130.5, 129.3, 124.7, 122.4, 119.2, 108.0, 75.7, 34.9, 26.2, 17.4, 17.2, 14.3, 14.3, 12.8, 9.7, 9.3 ppm. IR (neat): 2962, 2925, 1723, 1610, 1589, 1518, 1468, 1419, 1370, 1259, 1226, 1187, 1018, 921, 795, 748, 722, 678, 645, 605, 536 cm^–1^. HRMS (ESI-TOF) *m*/*z*: [M – H]^+^ Calcd for C_26_H_30_BF_2_N_3_O_2_ 464.2321; Found 464.2369.

##### Data for BODIPY **3aa**_**2**_ (Side Product)

Following the general procedure, chiral BODIPY **3aa**_**2**_ was obtained as an orange solid with an isolated yield of 16% after 4 days (side product, see Supporting Information file for the chemical structure**)**. ^1^H NMR (400 MHz, Chloroform-*d*) δ 7.28 (d, *J* = 7.8 Hz, 2H), 7.18 (d, *J* = 7.3 Hz, 2H), 7.12 (s, 1H), 6.99 (t, *J* = 7.5 Hz, 2H), 6.80 (d, *J* = 2.0 Hz, 2H), 6.33 (s, 2H), 3.51 (d, *J* = 15.1 Hz, 2H), 3.28 (d, *J* = 15.2 Hz, 2H), 3.18 (d, *J* = 5.7 Hz, 6H), 2.27 (s, 6H). ^13^C NMR (101 MHz, CDCl_3_) δ 177.1, 154.0, 154.0, 142.9, 141.5, 141.5, 133.4, 129.7, 129.7, 129.6, 124.4, 124.4, 122.8, 121.3, 120.2, 120.2, 108.3, 75.2, 36.7, 36.7, 26.2, 11.4 ppm. IR (neat): 3367, 2925, 1696, 1588, 1496, 1469, 1441, 1373, 1350, 1298, 1251, 1148, 1060, 978, 874, 828, 747, 696, 666, 580, 536, 479 cm^–1^. HRMS (ESI-TOF) *m*/*z*: [M + H]^+^ Calcd for C_31_H_29_BF_2_N_4_O_4_ 571.2328; Found 571.2372.

## Data Availability

The data underlying this study are available in the published article and its online Supporting Information.

## Supporting Information Available

Experimental details; characterization data; copies of NMR spectra; HRMS spectra, and chiral HPLC chromatograms (PDF)
