# Supplementary material for: Enantioselective Addition of 1,3,5,7-Tetramethyl-BODIPYs to Isatins by Bifunctional Quinine-Based Squaramides
Source: ACS Omega. 2025 Jan 1;10(1):1226–36. doi: 10.1021/acsomega.4c08792 (PMC11740631; doi:10.1021/acsomega.4c08792)
Supplement: Supplementary file 1 — ao4c08792_si_001.pdf [file ao4c08792_si_001.pdf]

Supporting Information

for

Enantioselective Addition of 1,3,5,7-tetramethyl-BODIPYs to Isatins by Bifunctional Quinine-Based Squaramides

Esra Dündar,[†] Murat Işık,[‡] Erol Yildirim,[†] and Cihangir Tanyeli*[,†]

[†]*Department of Chemistry, Middle East Technical University, 06800 Ankara, Türkiye. E-mail:* *tanyeli@metu.edu.tr*
[‡]*Department of Food Engineering, Bingöl University, 12000 Bingöl, Türkiye.*

Conformational analysis and Natural Transition Orbitals for 3aa **S2**

Copies of ^{1}H and ^{13}C NMR Spectra **S3**

Copies of HPLC Chromatogram **S13**

Copies of Mass Spectra **S22**

Conformational analysis and Natural Transition Orbitals for 3aa.

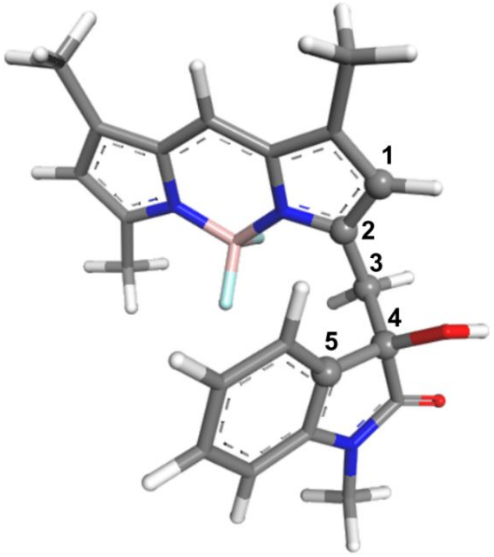

Figure S1. Conformational analysis was performed by classical methods using SciPCFF force field for two dihedral angles formed by atoms 1-2-3-4 and 2-3-4-5 to determine low energy structures which was optimized further by DFT methods to determine lowest energy structure.

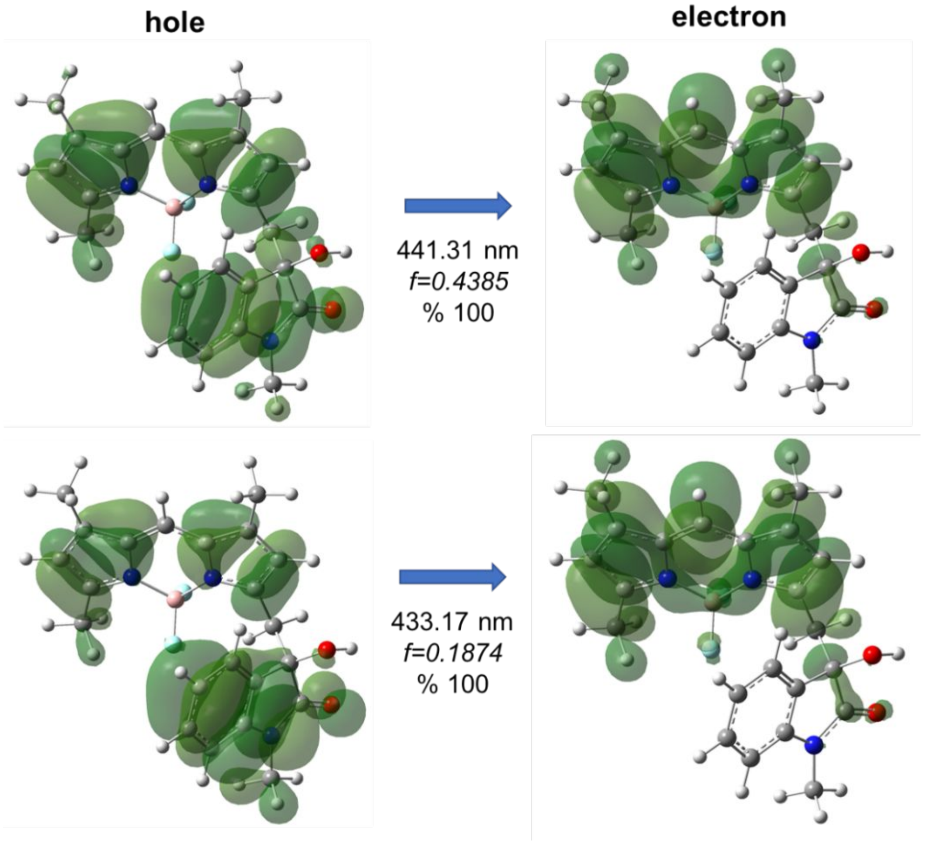

Figure S2. NTOs for the singlet excitations with highest oscillatory frequencies.

Copies of ^{1}H and ^{13}C NMR Spectra

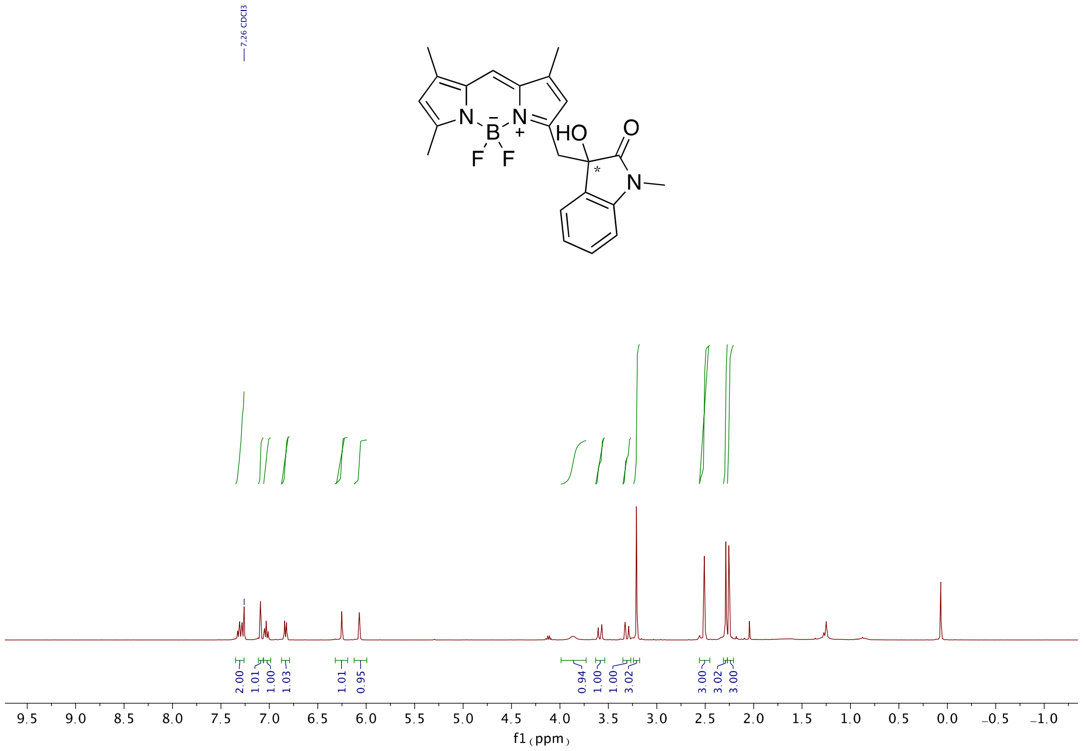

Figure S3. ^{1}H NMR spectrum (400 MHz) of compound **3aa** in $CDCl_3$ at rt.

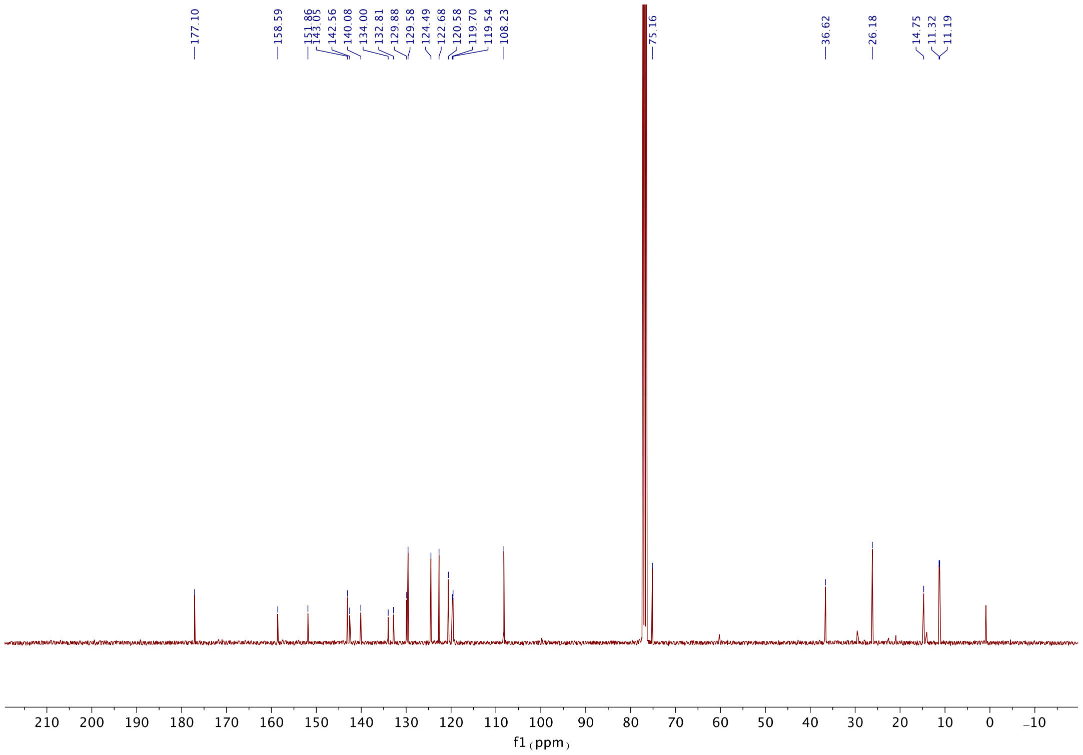

Figure S4. ^{13}C NMR spectrum (101 MHz) of compound **3aa** in $CDCl_3$ at rt.

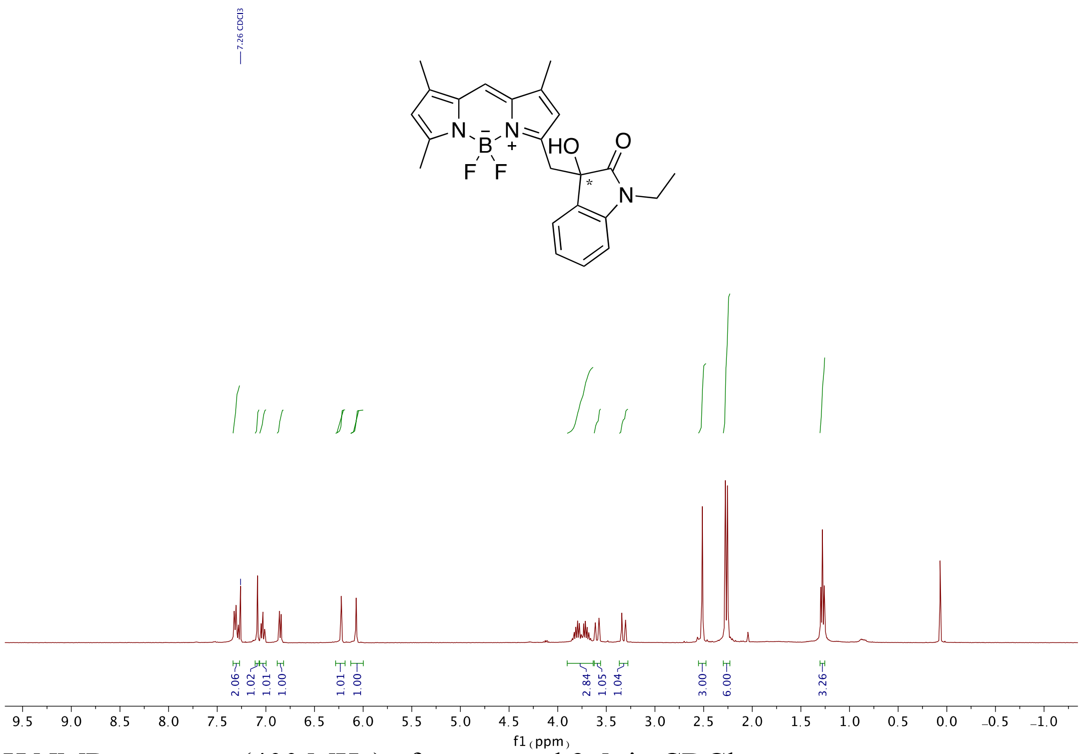

Figure S5. ^{1}H NMR spectrum (400 MHz) of compound **3ab** in $CDCl_3$ at rt.

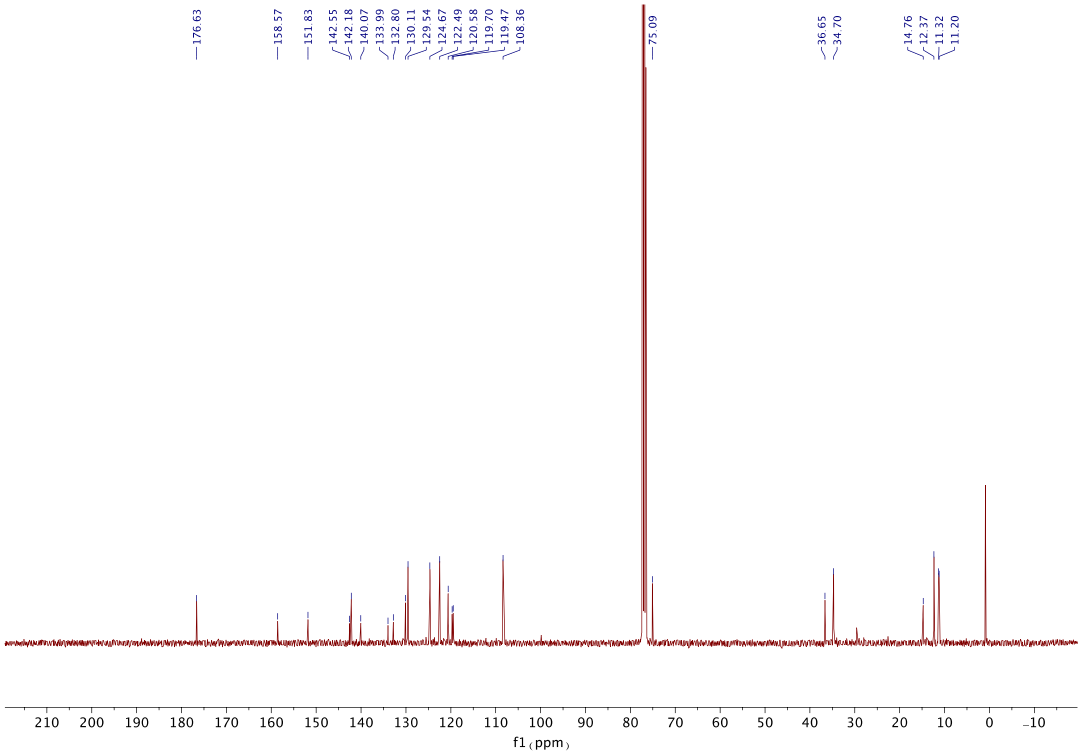

Figure S6. ^{13}C NMR spectrum (101 MHz) of compound **3ab** in $CDCl_3$ at rt.

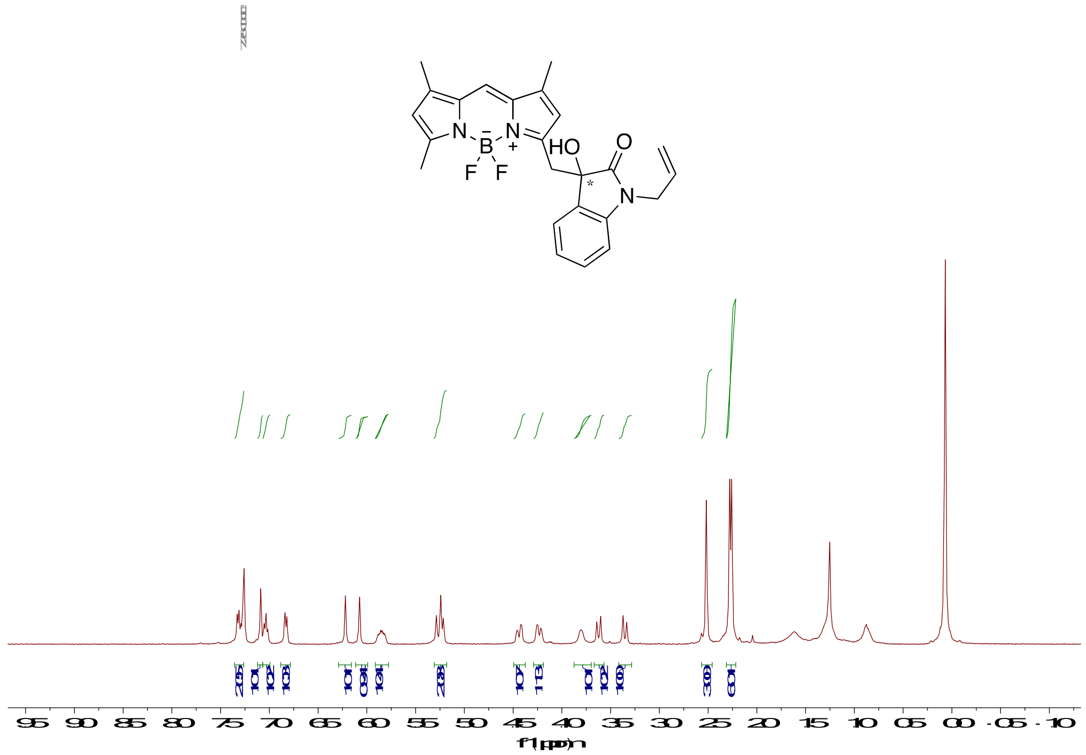

Figure S7. ^{1}H NMR spectrum (400 MHz) of compound **3ac** in $CDCl_3$ at rt.

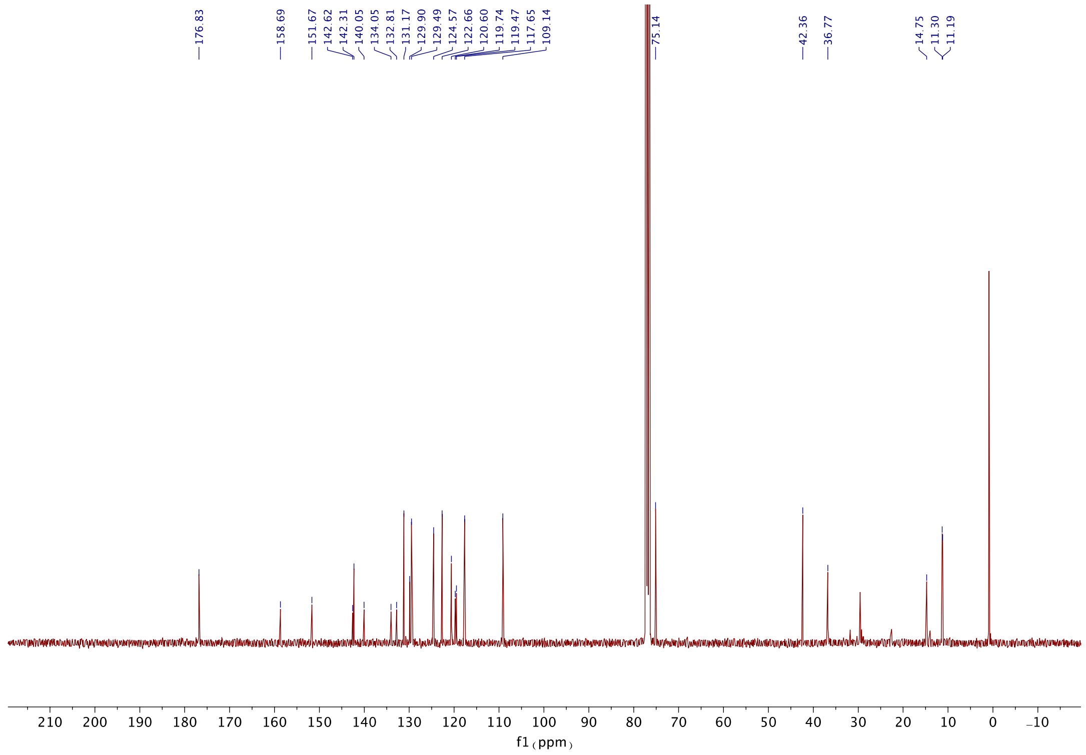

Figure S8. ^{13}C NMR spectrum (101 MHz) of compound **3ac** in $CDCl_3$ at rt.

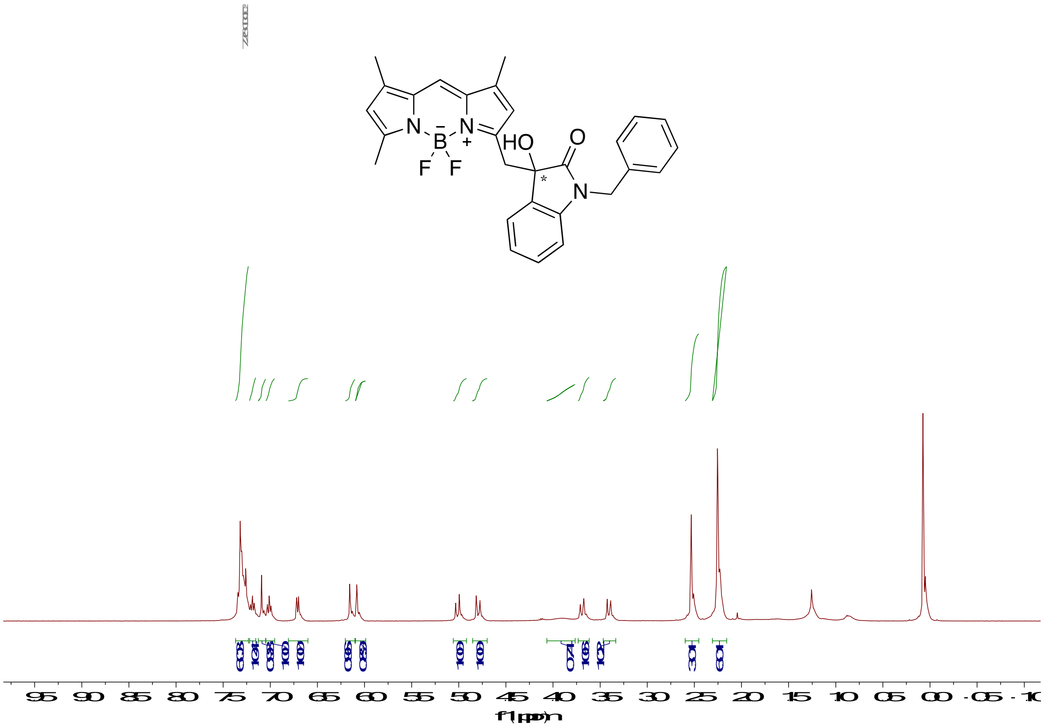

Figure S9. ^{1}H NMR spectrum (400 MHz) of compound **3ad** in $CDCl_3$ at rt.

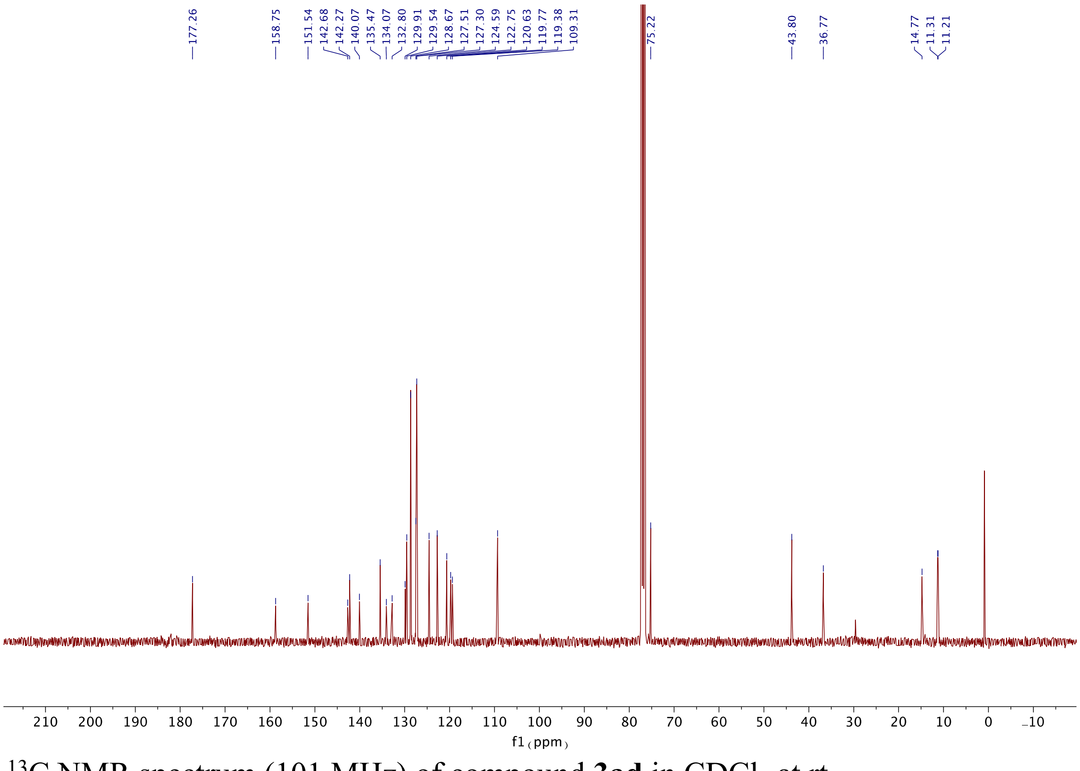

Figure S10. ^{13}C NMR spectrum (101 MHz) of compound **3ad** in $CDCl_3$ at rt.

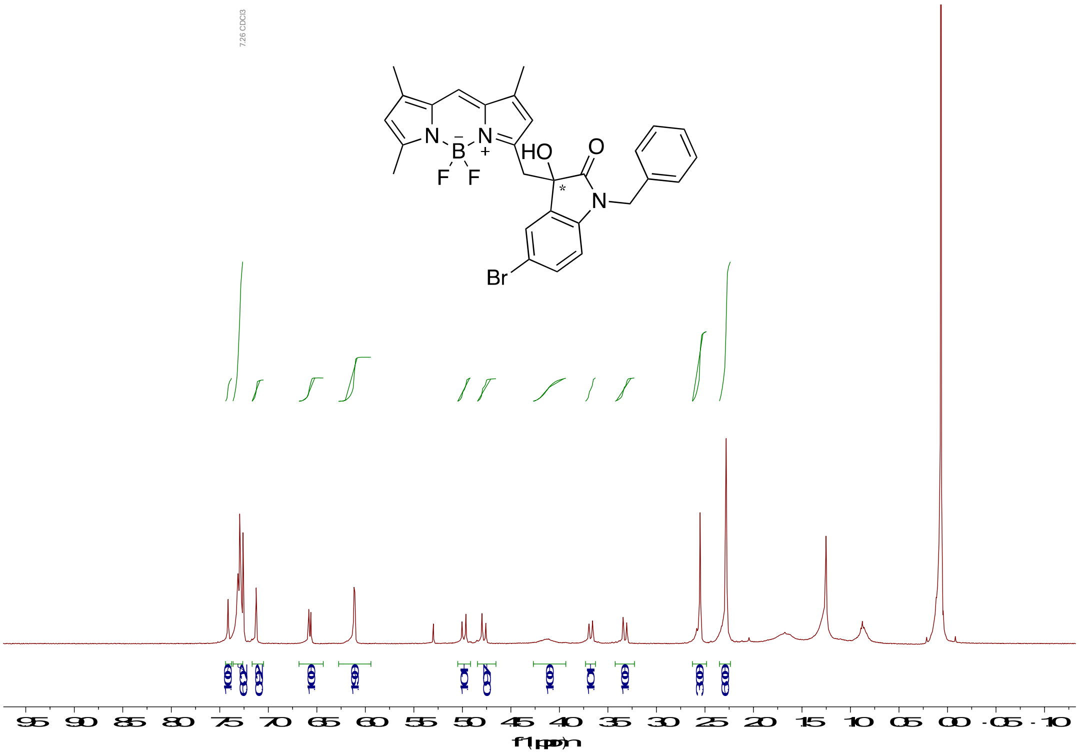

Figure S11. ^{1}H NMR spectrum (400 MHz) of compound **3ae** in $CDCl_3$ at rt.

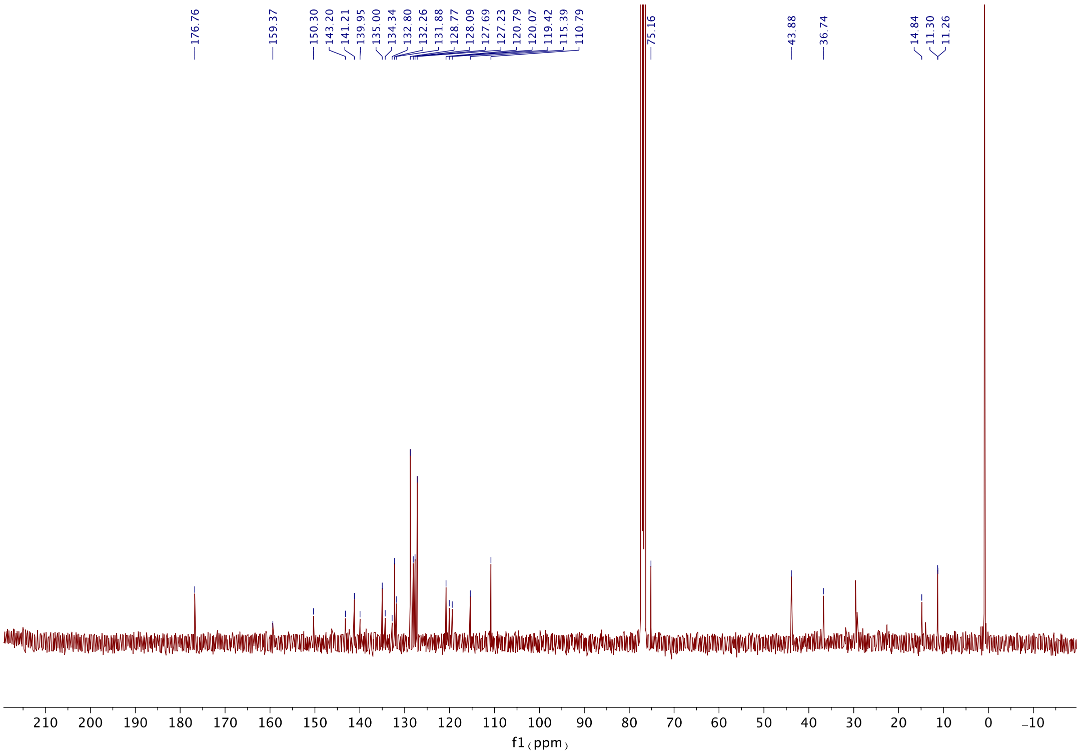

Figure S12. ^{13}C NMR spectrum (101 MHz) of compound **3ae** in $CDCl_3$ at rt.

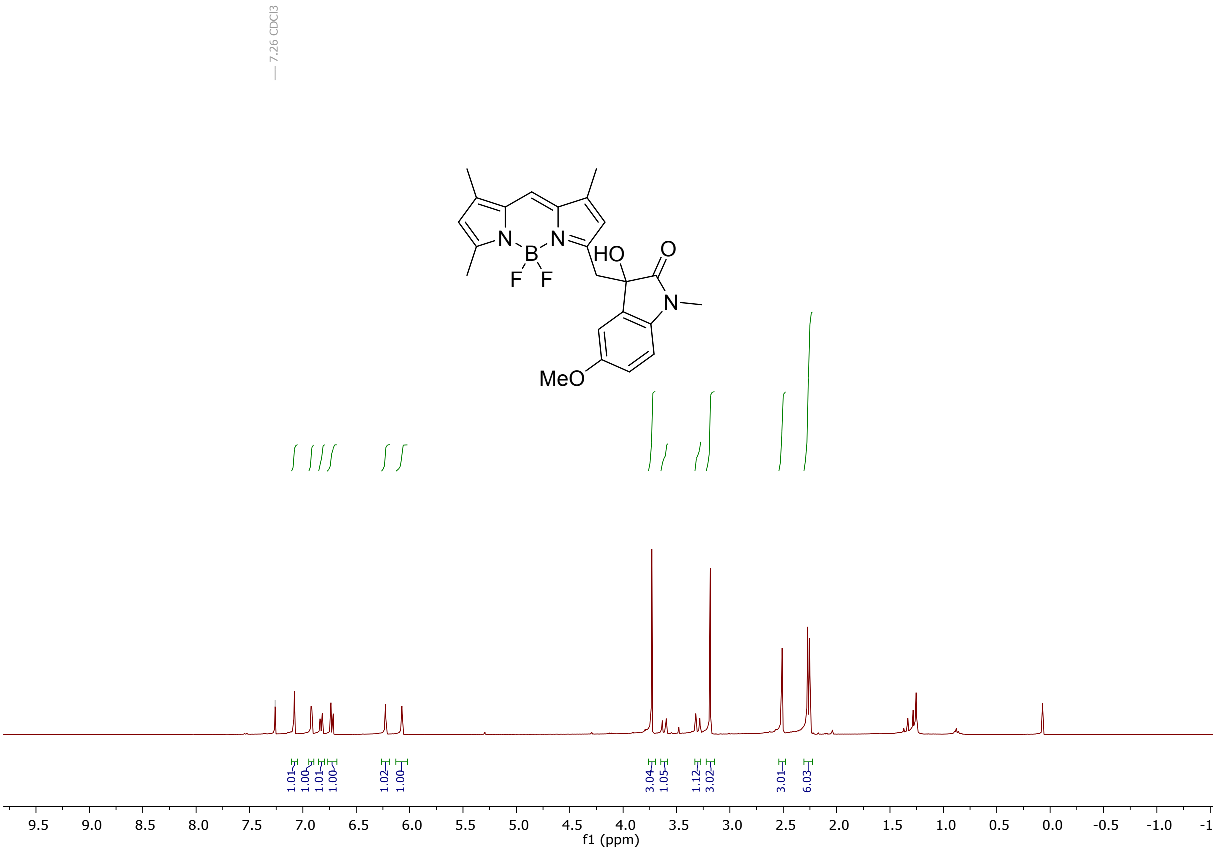

Figure S13. ^{1}H NMR spectrum (400 MHz) of compound **3ag** in $CDCl_3$ at rt.

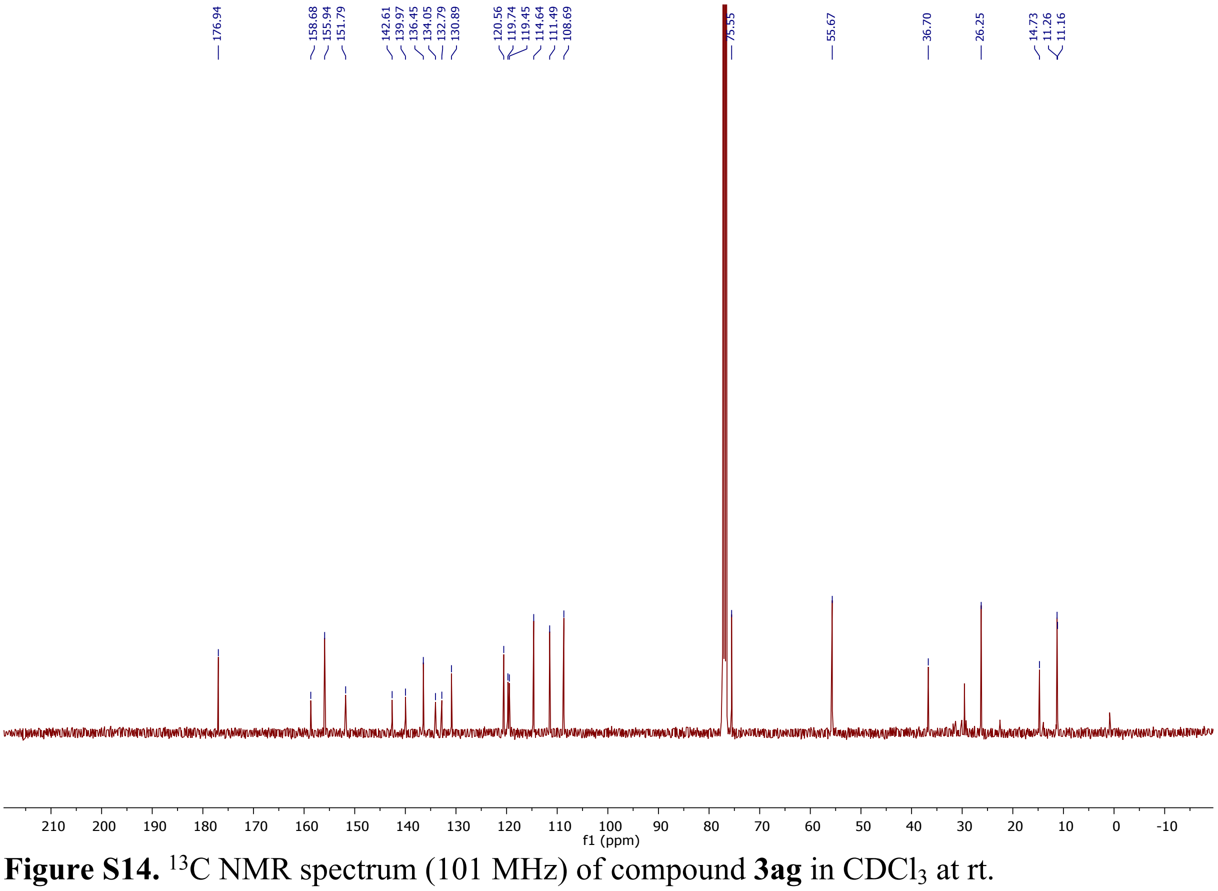

Figure S14. ^{13}C NMR spectrum (101 MHz) of compound **3ag** in $CDCl_3$ at rt.

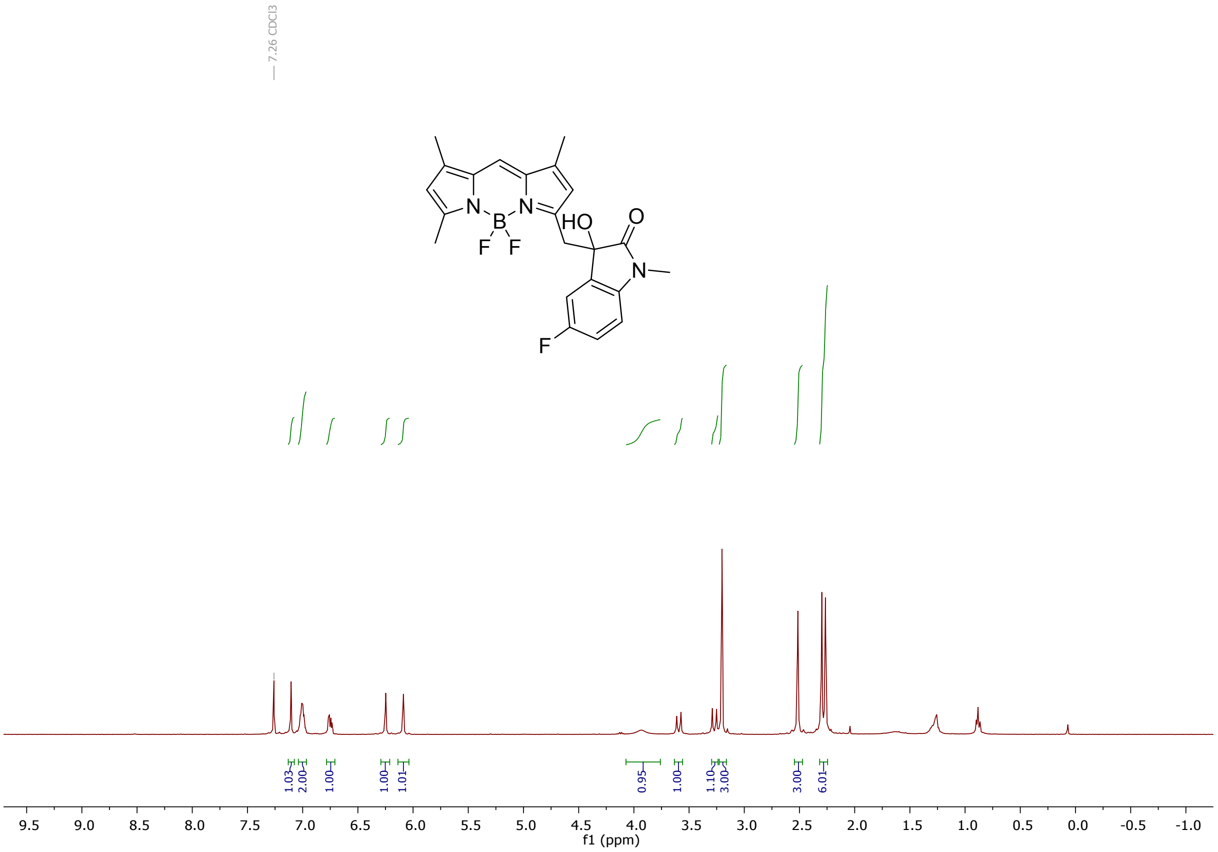

Figure S15. ^{1}H NMR spectrum (400 MHz) of compound **3af** in $CDCl_3$ at rt.

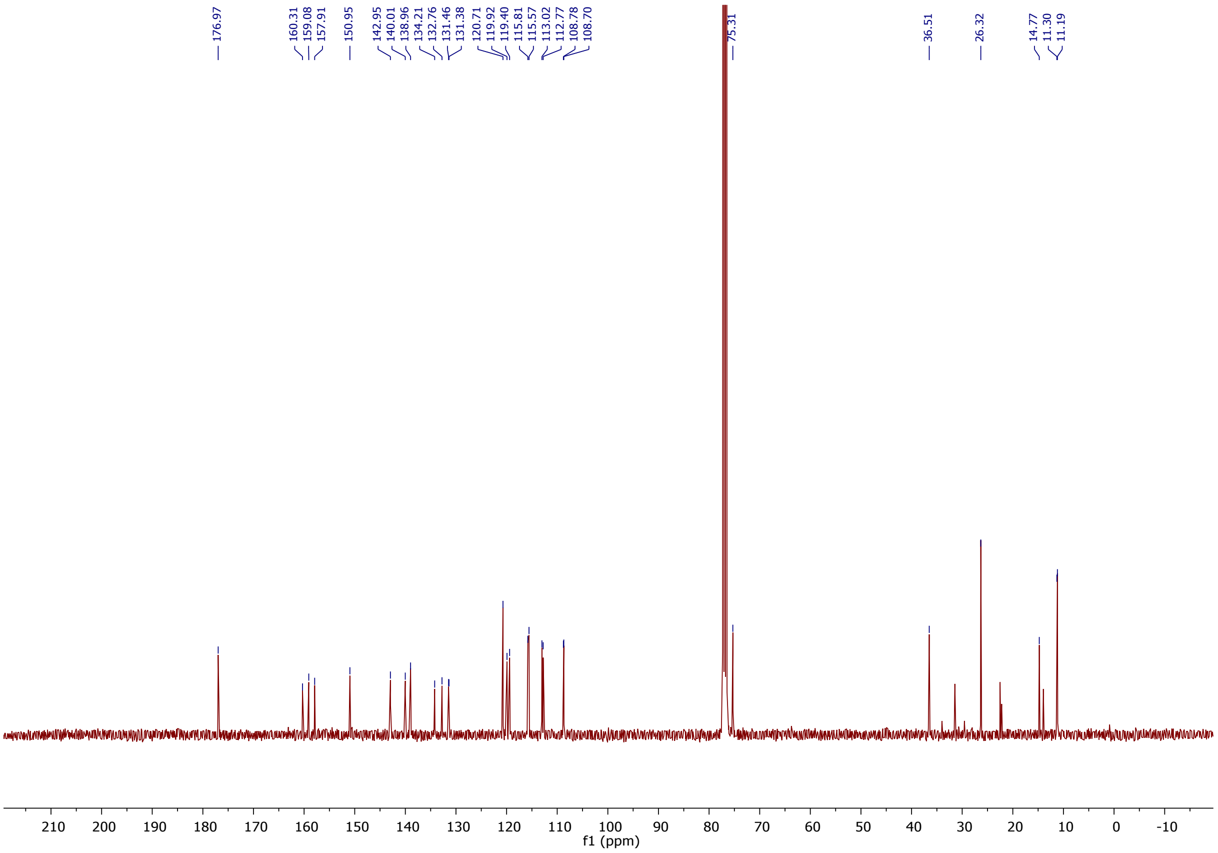

Figure S16. ^{13}C NMR spectrum (101 MHz) of compound **3af** in $CDCl_3$ at rt.

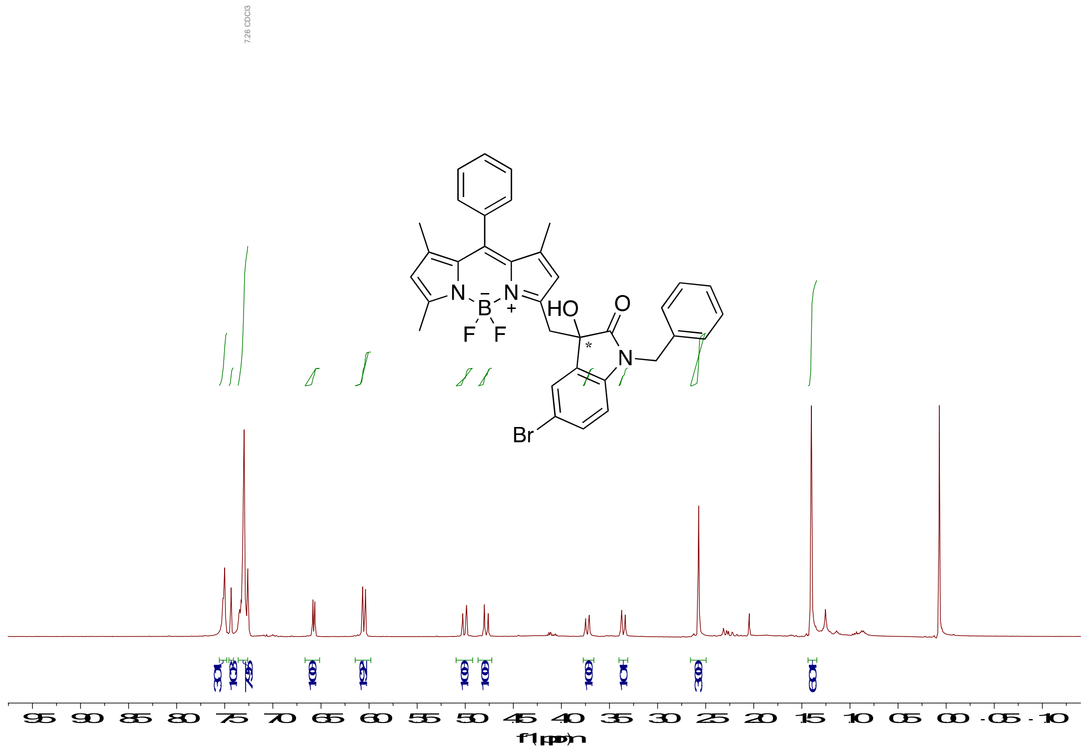

Figure S17. ^{1}H NMR spectrum (400 MHz) of compound **3be** in $CDCl_3$ at rt.

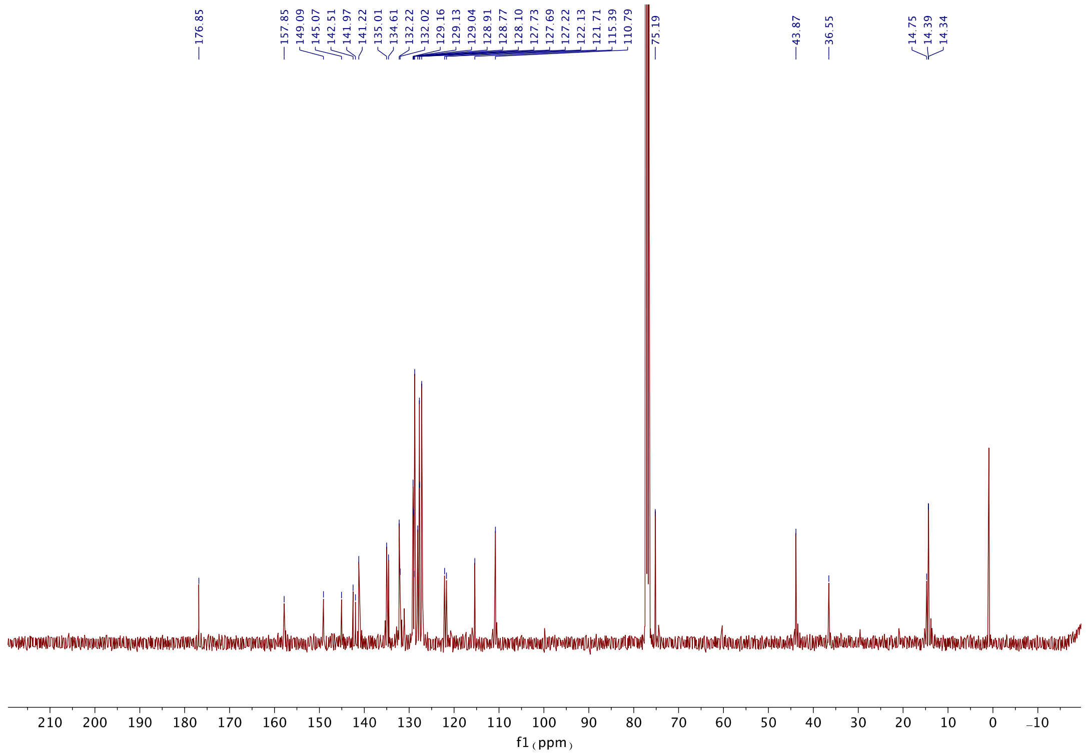

Figure S18. ^{13}C NMR spectrum (101 MHz) of compound **3be** in $CDCl_3$ at rt.

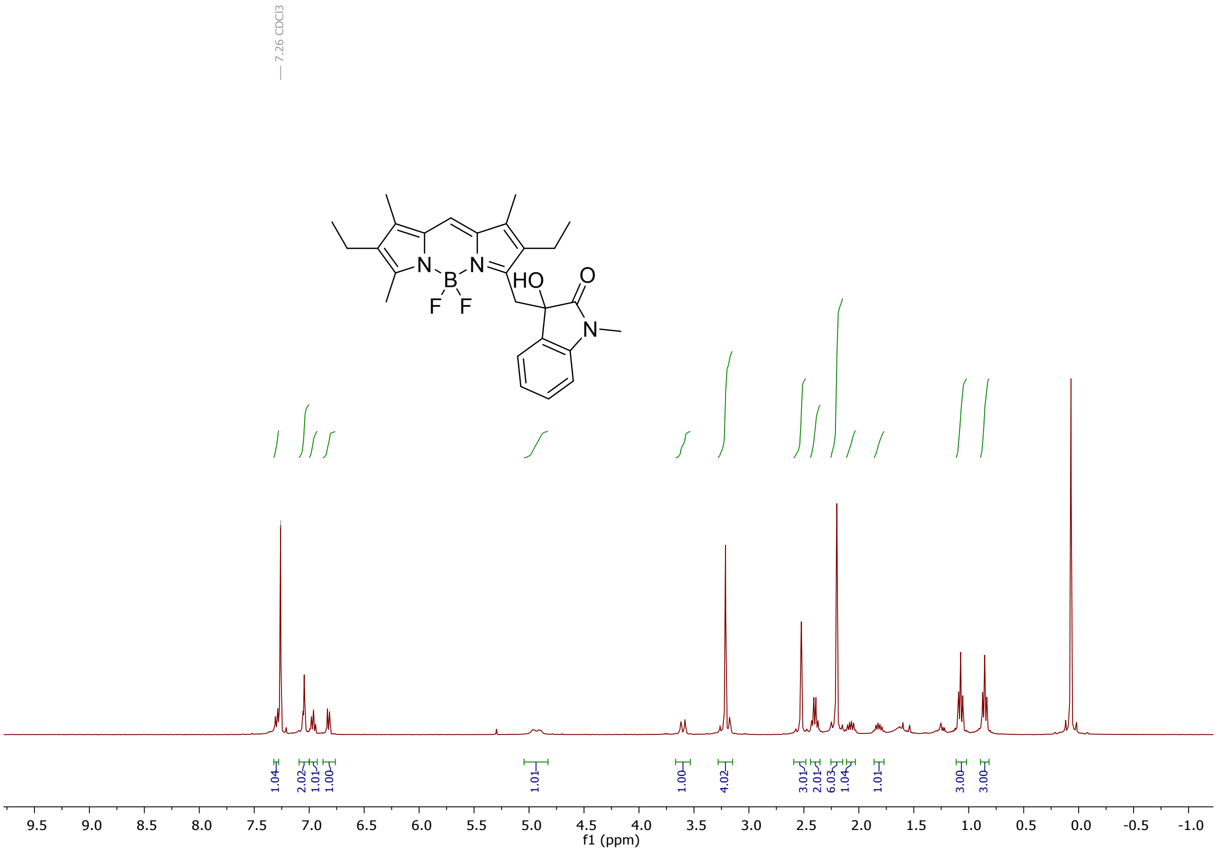

Figure S19. ^{1}H NMR spectrum (400 MHz) of compound **3ca** in $CDCl_3$ at rt.

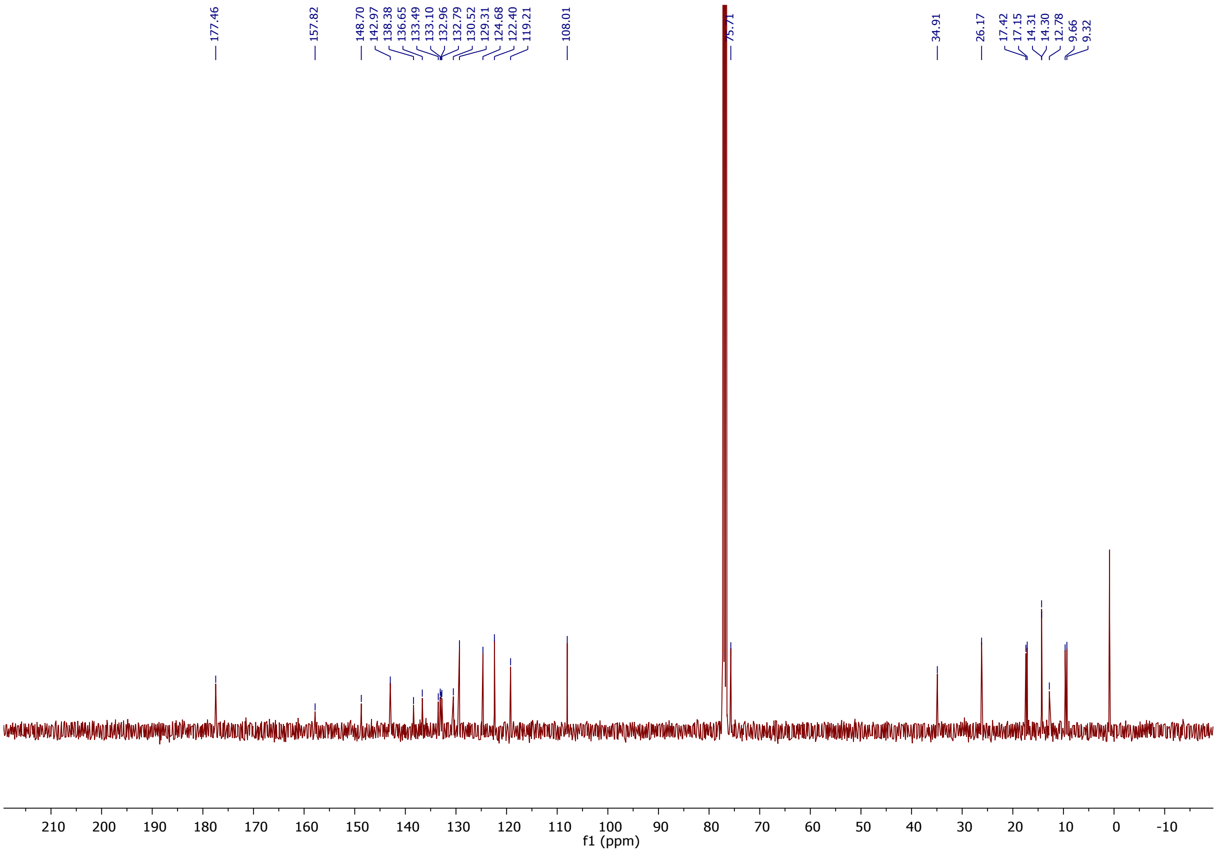

Figure S20. ^{13}C NMR spectrum (101 MHz) of compound **3ca** in $CDCl_3$ at rt.

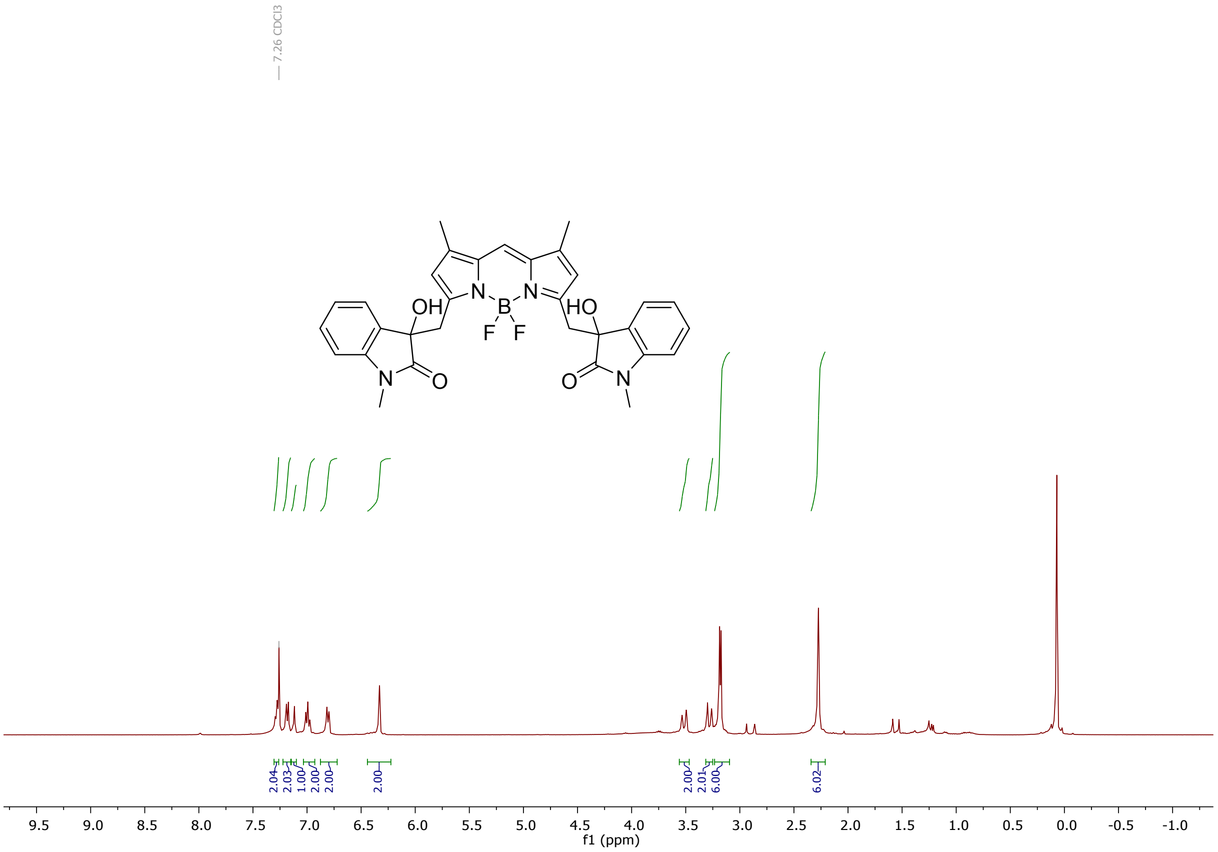

Figure S21. ^{1}H NMR spectrum (400 MHz) of compound **3aa$_2$** in $CDCl_3$ at rt.

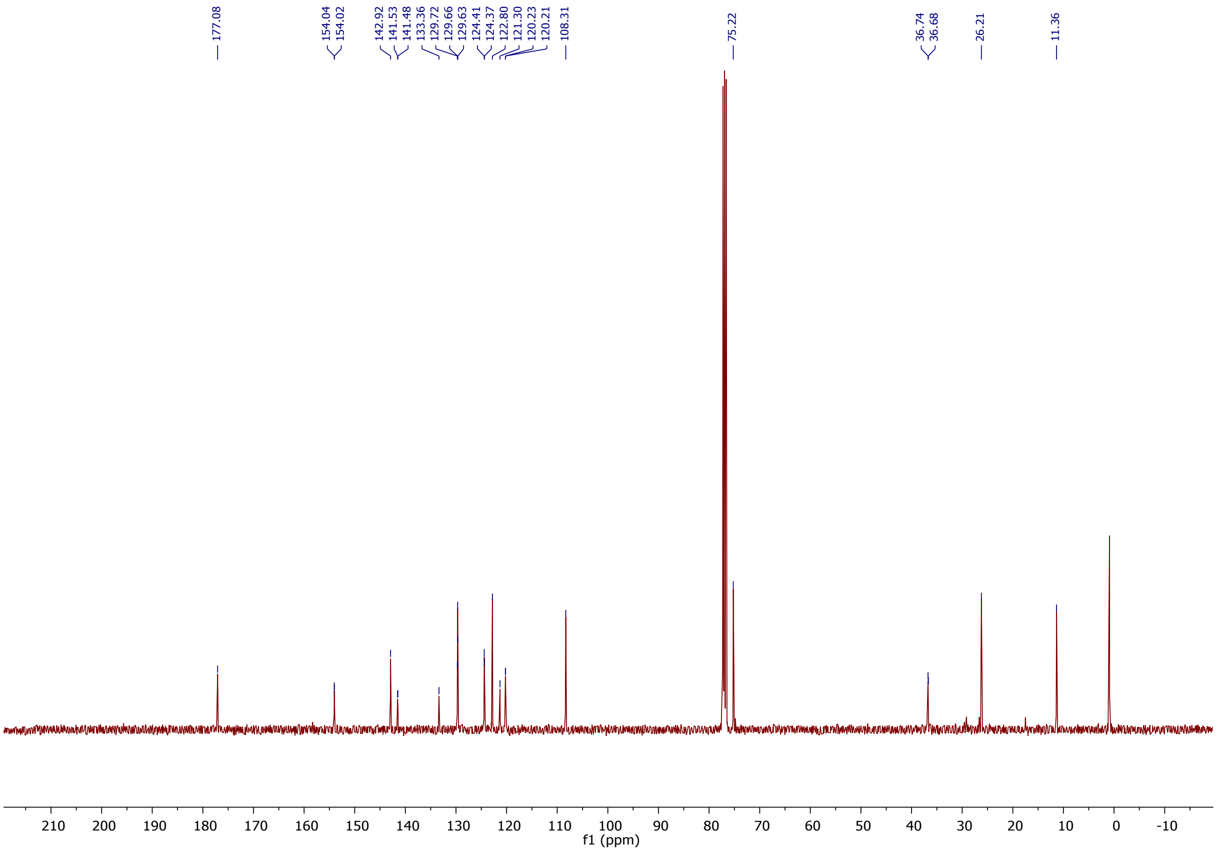

Figure S22. ^{13}C NMR spectrum (101 MHz) of compound **3aa$_2$** in $CDCl_3$ at rt.

Copies of HPLC Chromatogram

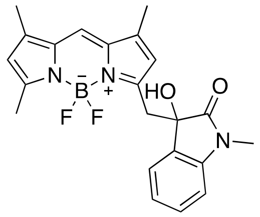

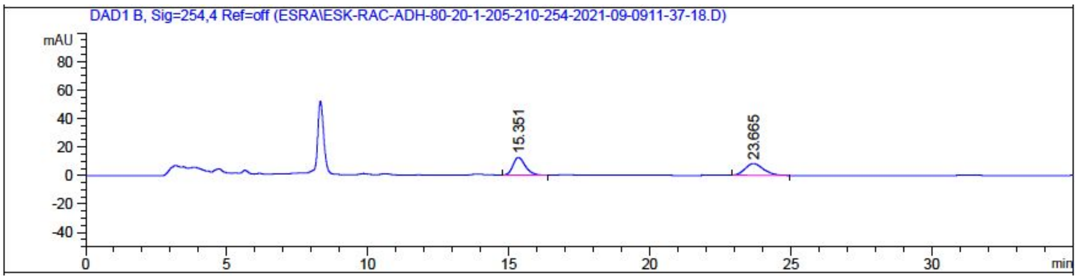

Signal 2: DAD1 B, Sig=254,4 Ref=off

Peak #	RetTime [min]	Type	Width [min]	Area [mAU*s]	Height [mAU]	Area %
1	15.351	BB	0.3580	377.56326	12.46240	50.9973
2	23.665	BB	0.5278	362.79602	8.05729	49.0027

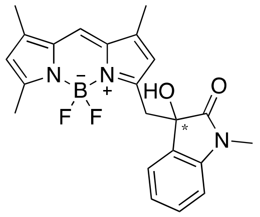

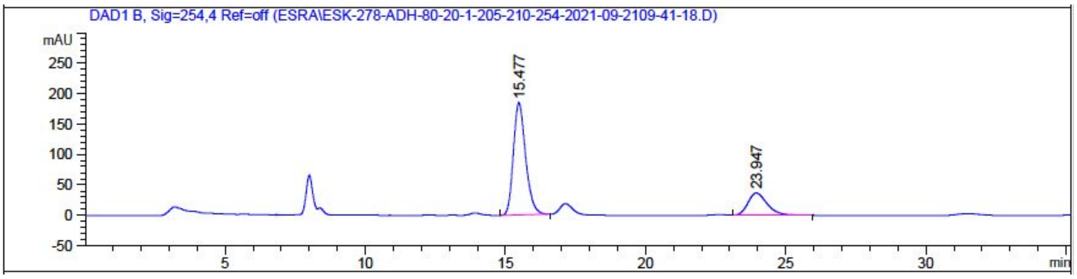

Signal 2: DAD1 B, Sig=254,4 Ref=off

Peak #	RetTime [min]	Type	Width [min]	Area [mAU*s]	Height [mAU]	Area %
1	15.477	BB	0.4598	5593.32861	184.18770	76.4617
2	23.947	BB	0.5762	1721.87231	36.31173	23.5383

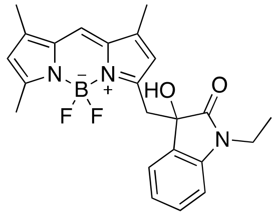

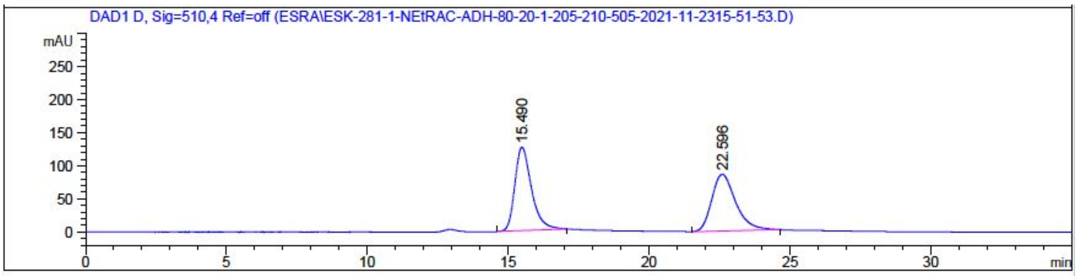

Signal 4: DAD1 D, Sig=510,4 Ref=off

Peak #	RetTime [min]	Type	Width [min]	Area [mAU*s]	Height [mAU]	Area %
1	15.490	VV R	0.4848	5202.46631	126.23048	50.7041
2	22.596	BV R	0.6960	5057.96924	85.77293	49.2959

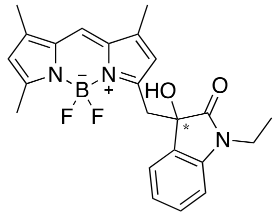

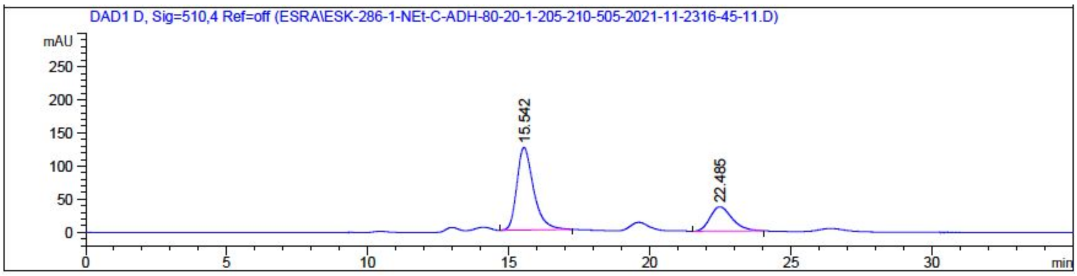

Signal 4: DAD1 D, Sig=510,4 Ref=off

Peak #	RetTime [min]	Type	Width [min]	Area [mAU*s]	Height [mAU]	Area %
1	15.542	VV R	0.5115	5153.97363	124.89008	72.1403
2	22.485	VV R	0.6364	1990.40210	36.74969	27.8597

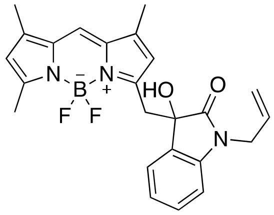

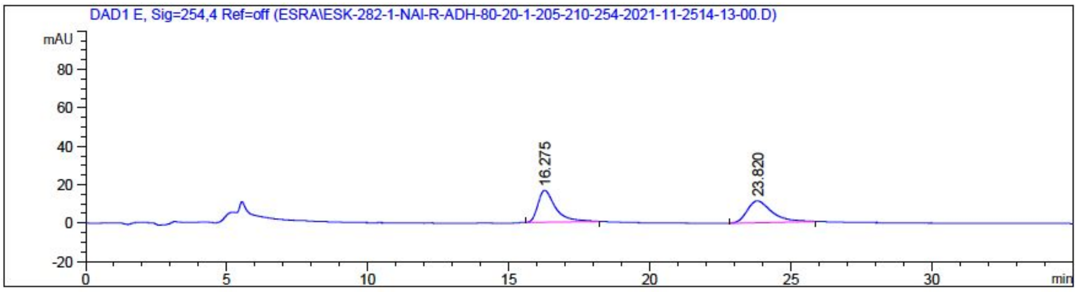

Signal 5: DAD1 E, Sig=254,4 Ref=off

Peak #	RetTime [min]	Type	Width [min]	Area [mAU*s]	Height [mAU]	Area %
1	16.275	BB	0.5173	727.18878	16.64234	51.3155
2	23.820	BB	0.7144	689.90491	11.32939	48.6845

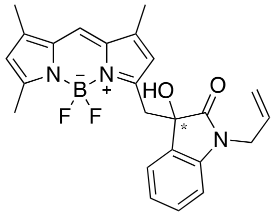

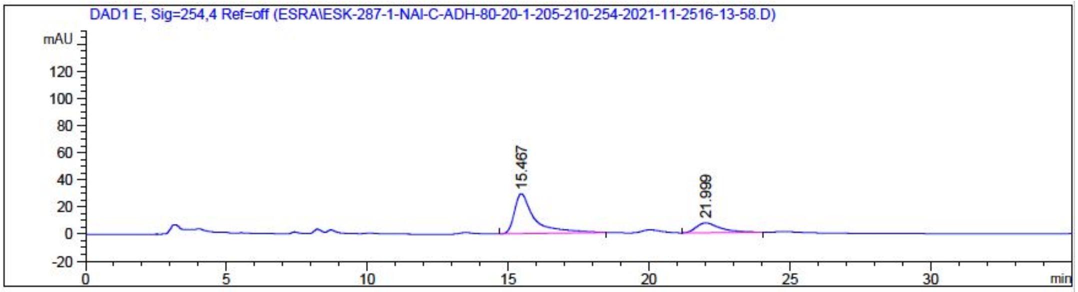

```
Signal 5: DAD1 E, Sig=254,4 Ref=off

Peak RetTime Type  Width     Area       Height     Area
  #   [min]        [min]    [mAU*s]     [mAU]       %
----|-------|----|-------|----------|----------|--------|
   1  15.467 BB    0.5982 1472.54126   29.55941  77.6944
   2  21.999 BB    0.6894  422.75748    7.17993  22.3056
```

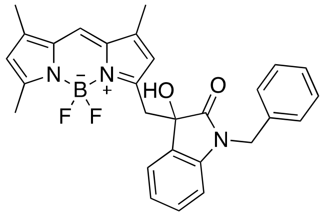

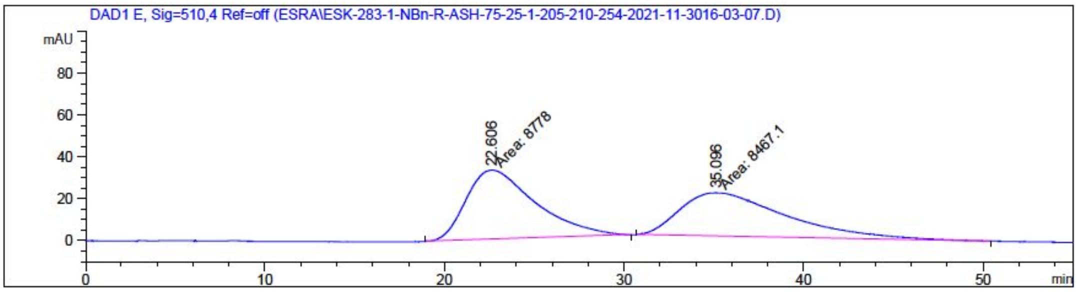


```
Signal 5: DAD1 E, Sig=510,4 Ref=off

Peak RetTime Type  Width     Area       Height     Area
  #   [min]        [min]    [mAU*s]     [mAU]       %
----|-------|----|-------|----------|----------|--------|
   1  22.606 MM    4.4330 8778.00293   33.00220  50.9014
   2  35.096 MM    6.8287 8467.09668   20.66540  49.0986
```

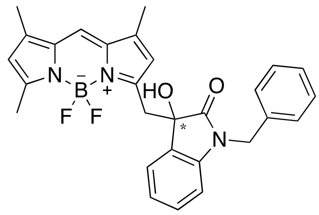

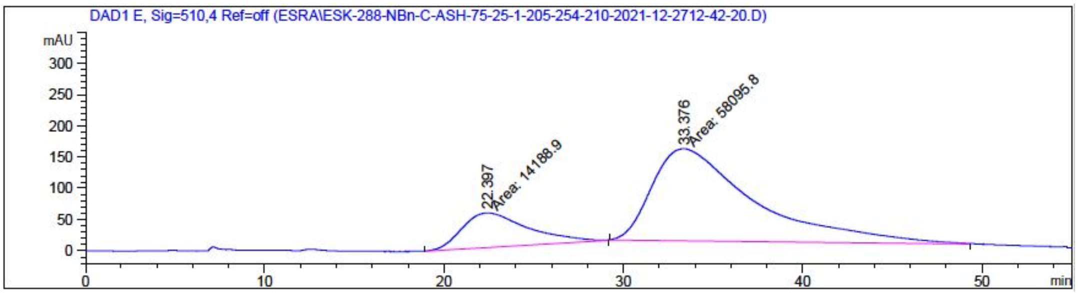

Signal 5: DAD1 E, Sig=510,4 Ref=off

Peak #	RetTime [min]	Type	Width [min]	Area [mAU*s]	Height [mAU]	Area %
1	22.397	MM	4.2453	1.41889e4	55.70435	19.6292
2	33.376	MM	6.5680	5.80958e4	147.42049	80.3708

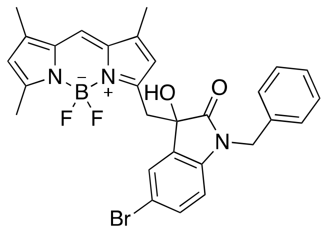

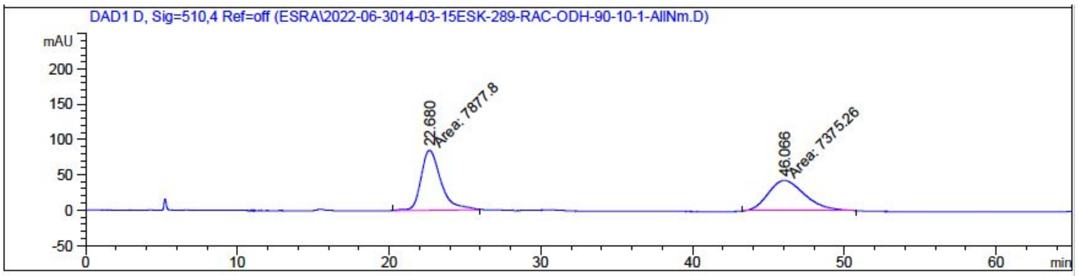

Signal 4: DAD1 D, Sig=510,4 Ref=off

Peak #	RetTime [min]	Type	Width [min]	Area [mAU*s]	Height [mAU]	Area %
1	22.680	MM	1.5370	7877.80420	85.42406	51.6473
2	46.066	MM	2.8459	7375.26367	43.19309	48.3527

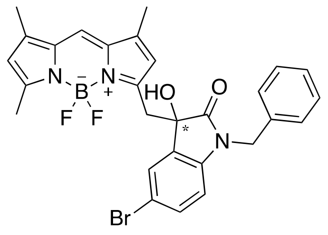

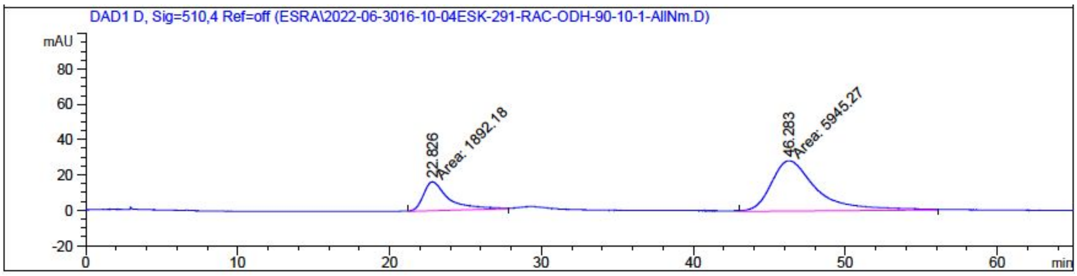

Signal 4: DAD1 D, Sig=510,4 Ref=off

Peak #	RetTime [min]	Type	Width [min]	Area [mAU*s]	Height [mAU]	Area %
1	22.826	MM	1.9267	1892.18140	16.36839	24.1428
2	46.283	MM	3.4824	5945.27148	28.45380	75.8572

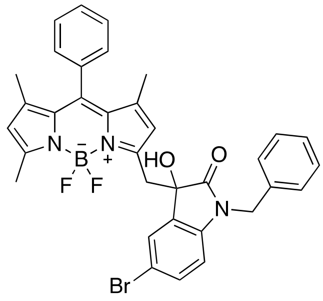

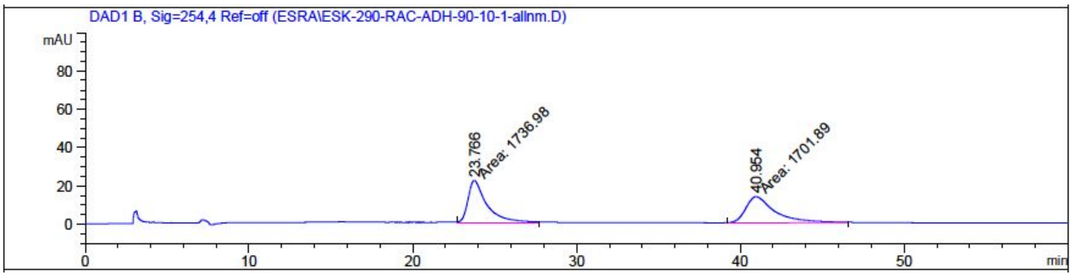

Signal 2: DAD1 B, Sig=254,4 Ref=off

Peak #	RetTime [min]	Type	Width [min]	Area [mAU*s]	Height [mAU]	Area %
1	23.766	MM	1.3273	1736.98438	21.81134	50.5103
2	40.954	MM	2.0686	1701.88831	13.71236	49.4897

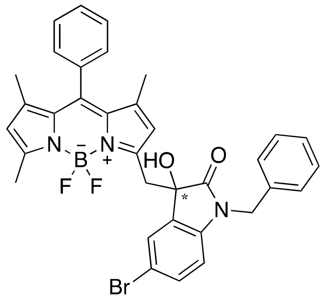

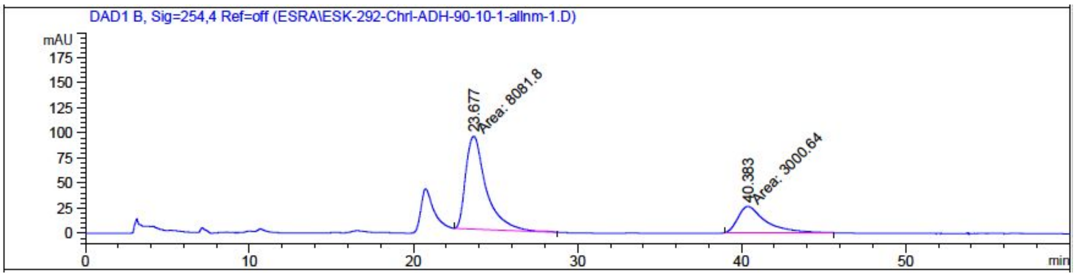

Signal 2: DAD1 B, Sig=254,4 Ref=off

Peak #	RetTime [min]	Type	Width [min]	Area [mAU*s]	Height [mAU]	Area %
1	23.677	MM	1.4579	8081.79834	92.39211	72.9244
2	40.383	MM	1.9298	3000.63843	25.91506	27.0756

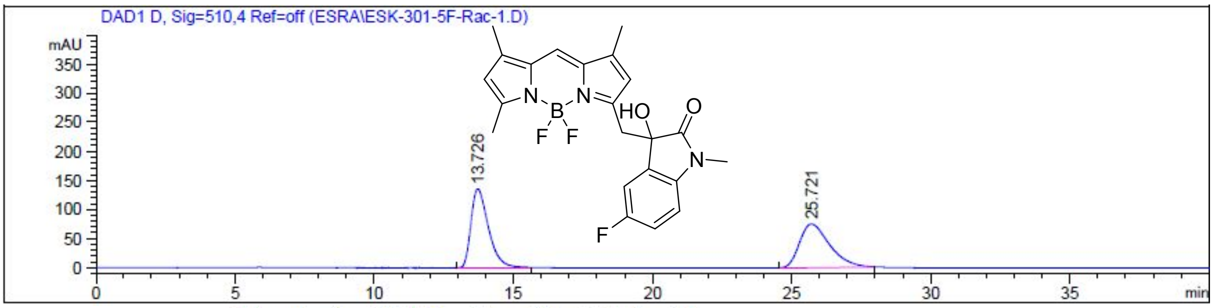

Signal 4: DAD1 D, Sig=510,4 Ref=off

Peak #	RetTime [min]	Type	Width [min]	Area [mAU*s]	Height [mAU]	Area %
1	13.726	BV R	0.5143	5890.79688	135.75107	51.1543
2	25.721	BB	0.8790	5624.94189	74.91307	48.8457

Totals : 1.15157e4 210.66414

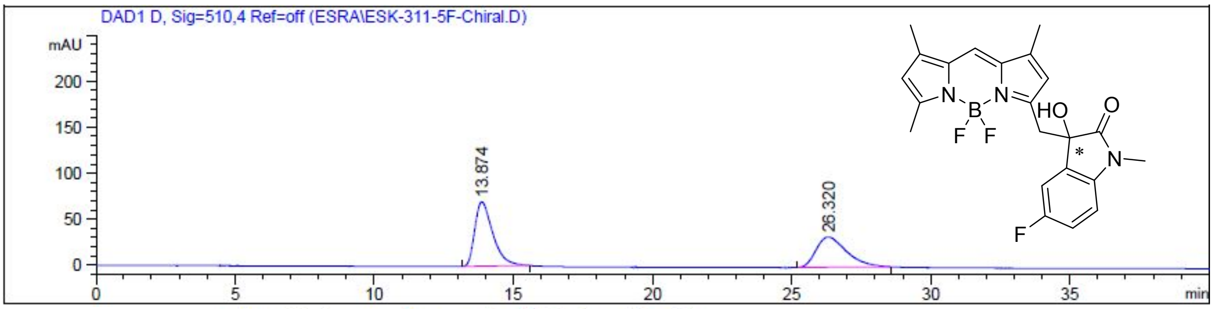

Signal 4: DAD1 D, Sig=510,4 Ref=off

Peak #	RetTime [min]	Type	Width [min]	Area [mAU*s]	Height [mAU]	Area %
1	13.874	BB	0.5346	3148.80420	69.90019	55.7844
2	26.320	BB	0.8929	2495.78735	32.71955	44.2156

Totals : 5644.59155 102.61974

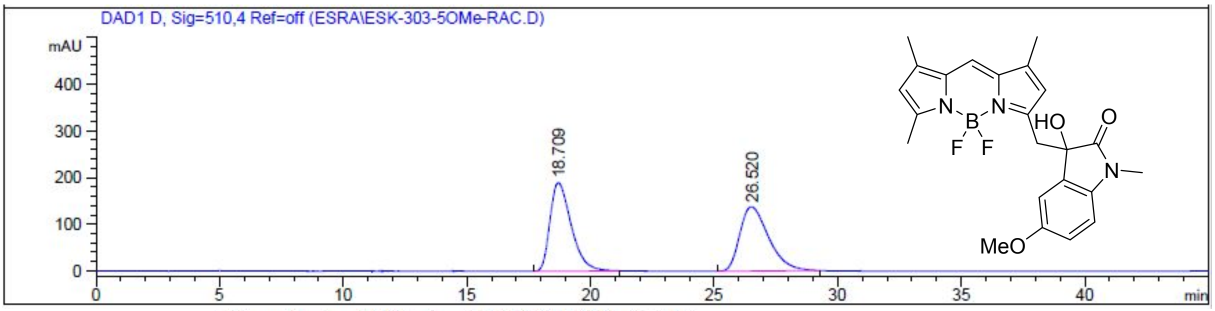

Signal 4: DAD1 D, Sig=510,4 Ref=off

Peak #	RetTime [min]	Type	Width [min]	Area [mAU*s]	Height [mAU]	Area %
1	18.709	BB	0.6992	1.12486e4	188.64272	50.2710
2	26.520	BB	0.9477	1.11273e4	137.34102	49.7290
Totals :				2.23759e4	325.98373	

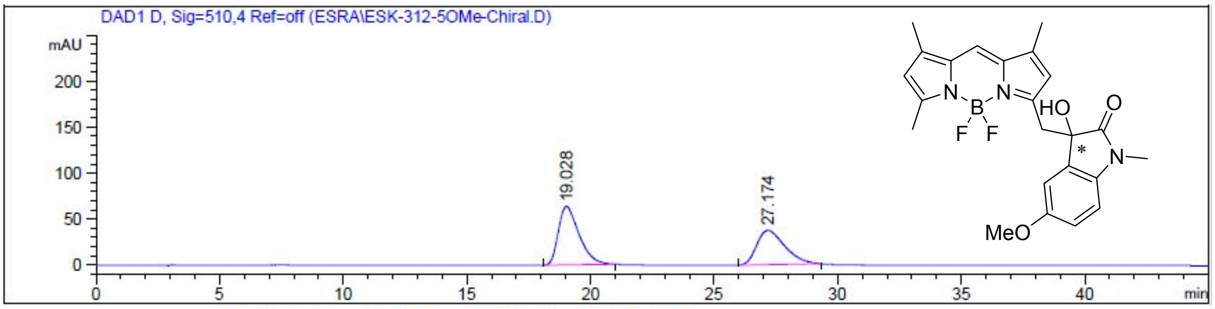

Signal 4: DAD1 D, Sig=510,4 Ref=off

Peak #	RetTime [min]	Type	Width [min]	Area [mAU*s]	Height [mAU]	Area %
1	19.028	BB	0.6823	3695.88013	63.47847	55.5847
2	27.174	BB	0.9265	2953.22144	37.30671	44.4153
Totals :				6649.10156	100.78518	

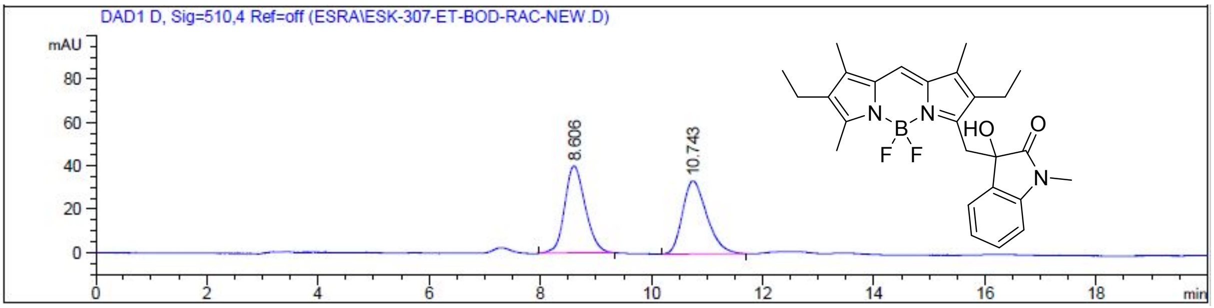

Signal 4: DAD1 D, Sig=510,4 Ref=off

Peak #	RetTime [min]	Type	Width [min]	Area [mAU*s]	Height [mAU]	Area %
1	8.606	BB	0.3161	1034.12561	40.20695	50.1612
2	10.743	BB	0.3606	1027.47803	33.61668	49.8388
Totals :				2061.60364	73.82363	

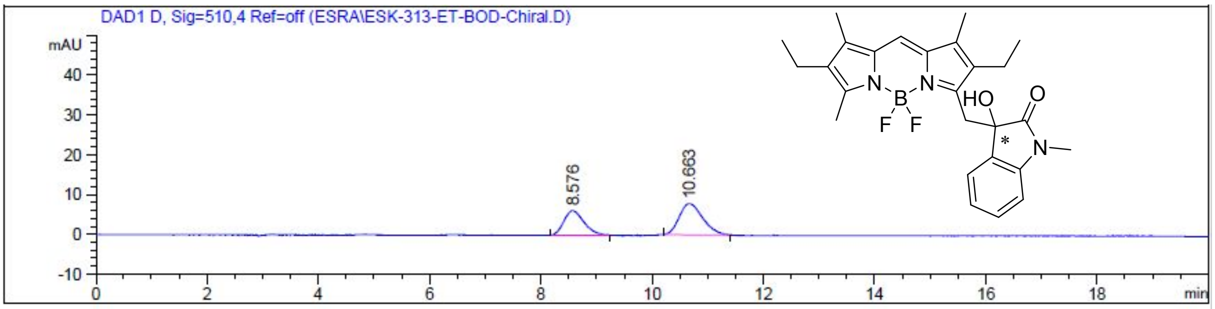

Signal 4: DAD1 D, Sig=510,4 Ref=off

Peak #	RetTime [min]	Type	Width [min]	Area [mAU*s]	Height [mAU]	Area %
1	8.576	BB	0.2933	152.66881	6.13509	40.0412
2	10.663	BB	0.3439	228.61095	7.82386	59.9588
Totals :				381.27975	13.95895	

HPLC Chromatograms of Kinetic Resolution for *rac*-3aa

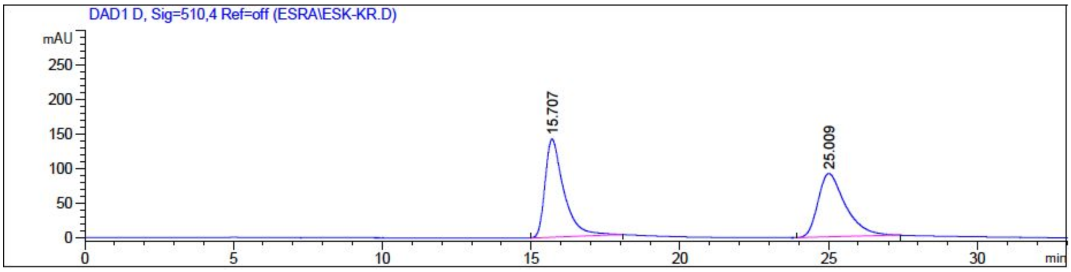

Signal 4: DAD1 D, Sig=510,4 Ref=off

Peak #	RetTime [min]	Type	Width [min]	Area [mAU*s]	Height [mAU]	Area %
1	15.707	BB	0.5427	6250.54834	142.22469	51.3828
2	25.009	BB	0.7587	5914.12842	91.97034	48.6172
Totals :				1.21647e4	234.19502	

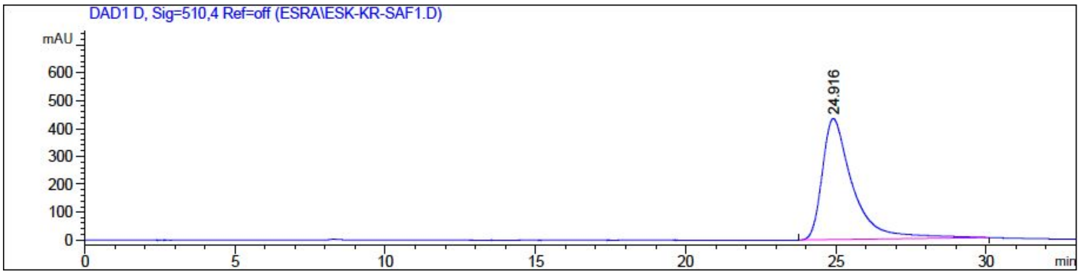

Signal 4: DAD1 D, Sig=510,4 Ref=off

Peak #	RetTime [min]	Type	Width [min]	Area [mAU*s]	Height [mAU]	Area %
1	24.916	BB	0.8310	3.07572e4	433.89075	100.0000

Totals : 3.07572e4 433.89075

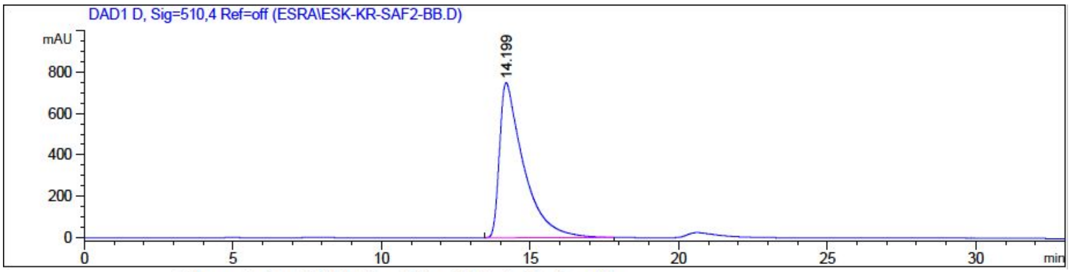

Signal 4: DAD1 D, Sig=510,4 Ref=off

Peak #	RetTime [min]	Type	Width [min]	Area [mAU*s]	Height [mAU]	Area %
1	14.199	BB	0.7354	4.21835e4	749.22467	100.0000

Totals : 4.21835e4 749.22467

Copies of Mass Spectra

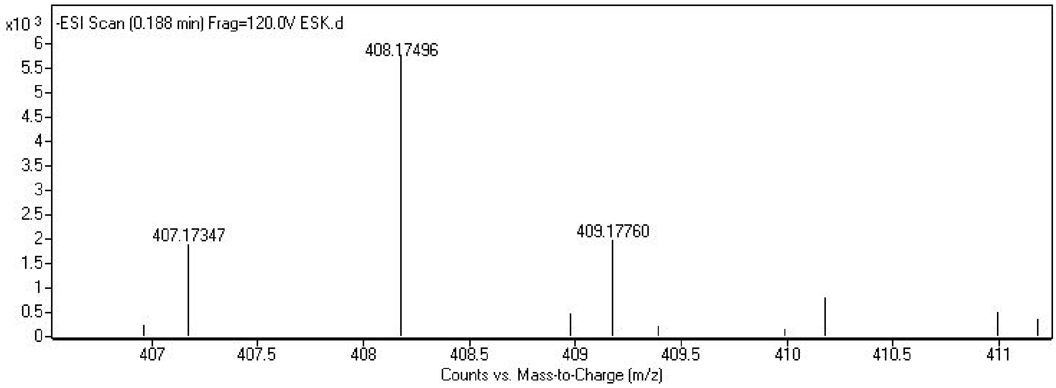

Figure S23. HRMS spectrum of **3aa**.

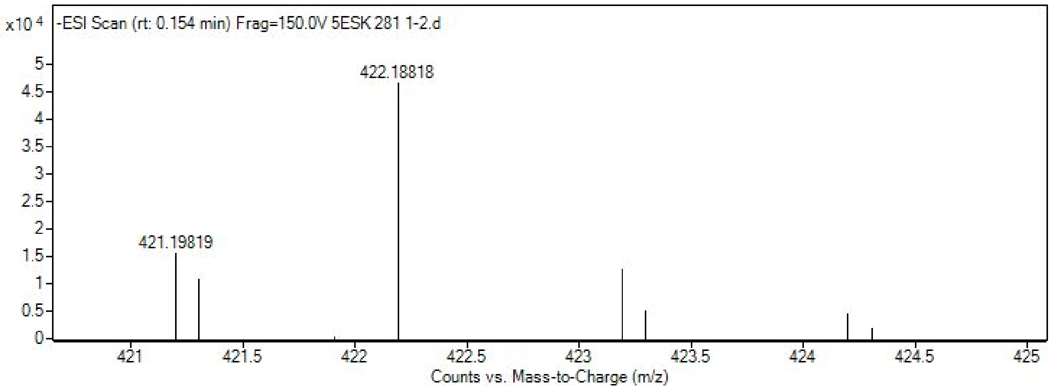

Figure S24. HRMS spectrum of **3ab**.

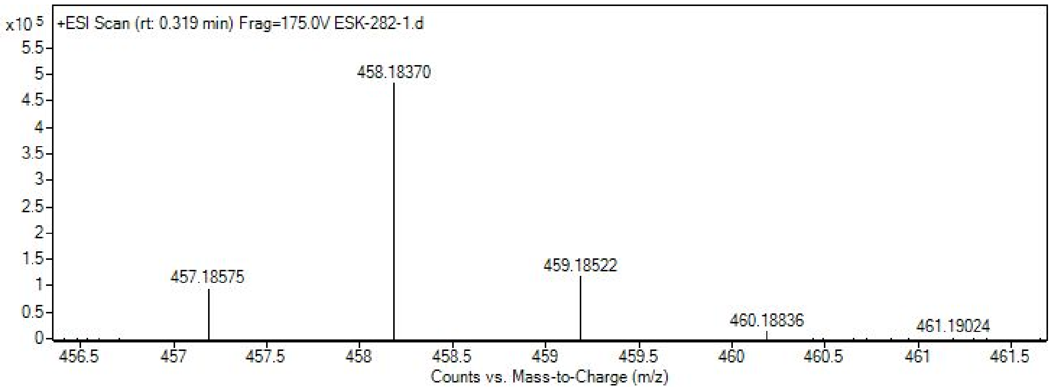

Figure S25. HRMS spectrum of **3ac**.

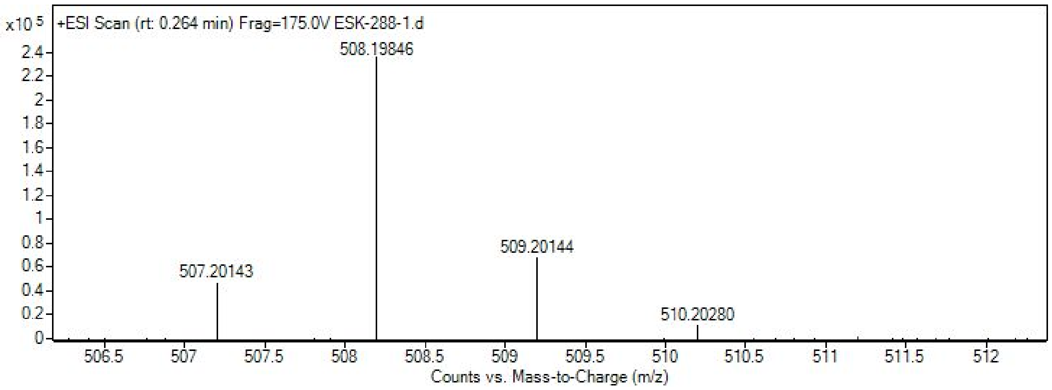

Figure S26. HRMS spectrum of **3ad**.

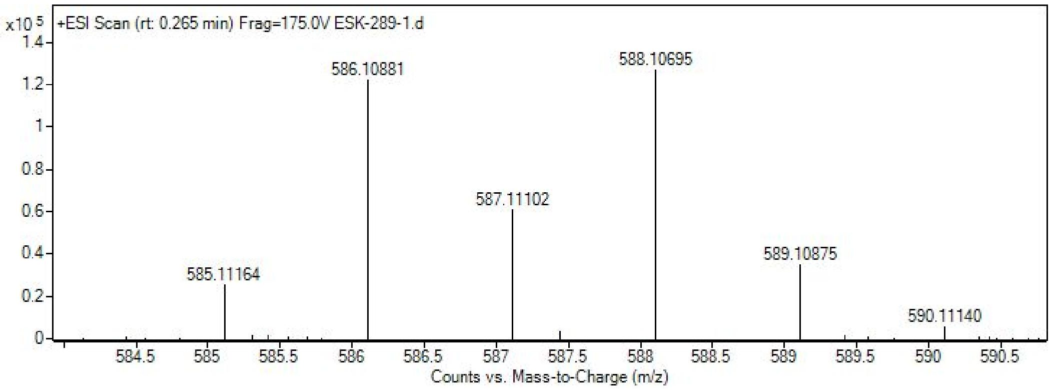

Figure S27. HRMS spectrum of **3ae**.

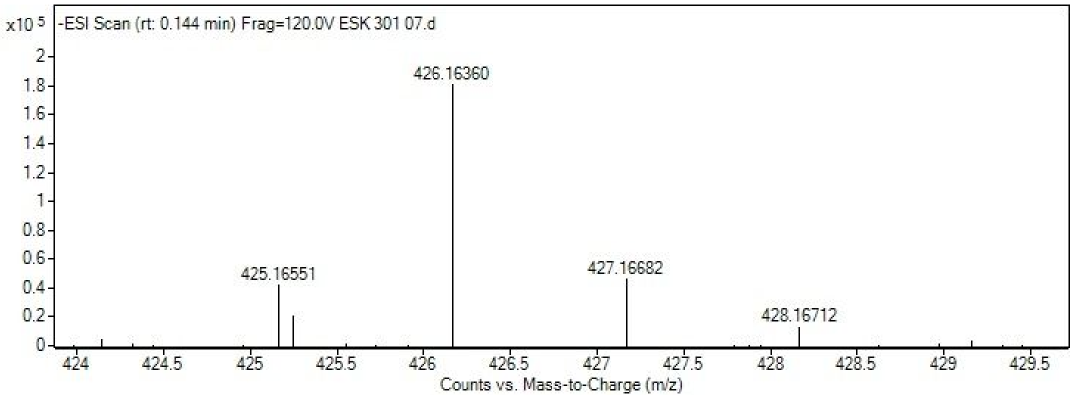

Figure S28. HRMS spectrum of **3af**.

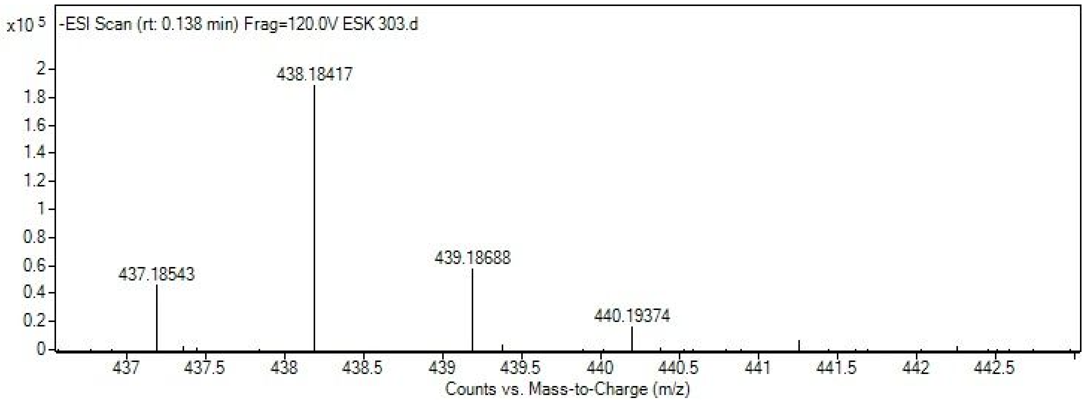

Figure S29. HRMS spectrum of **3ag**.

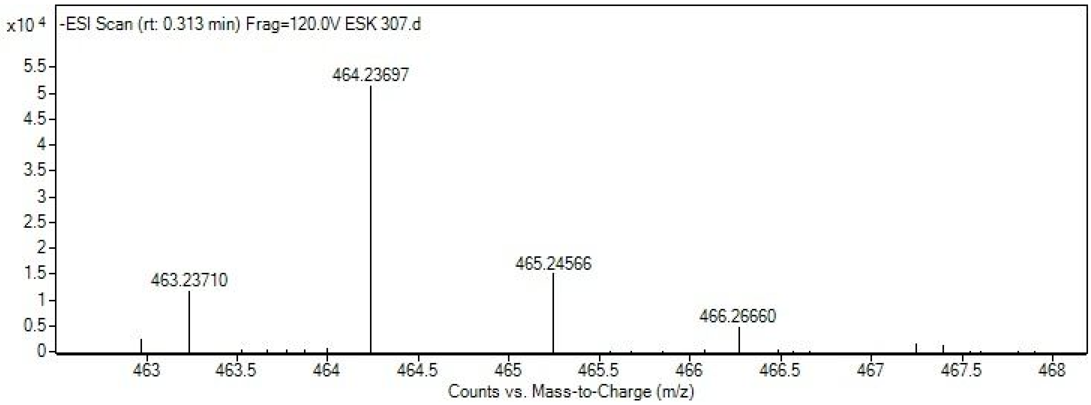

Figure S30. HRMS spectrum of **3ca**.

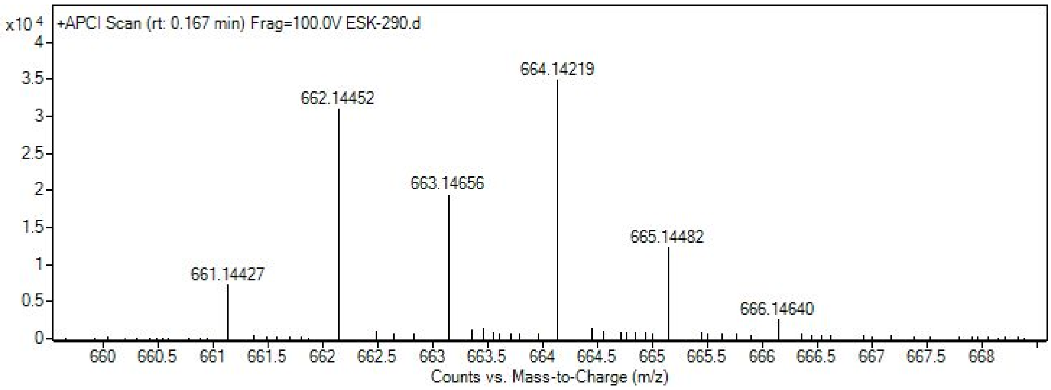

Figure S31. HRMS spectrum of **3be**.

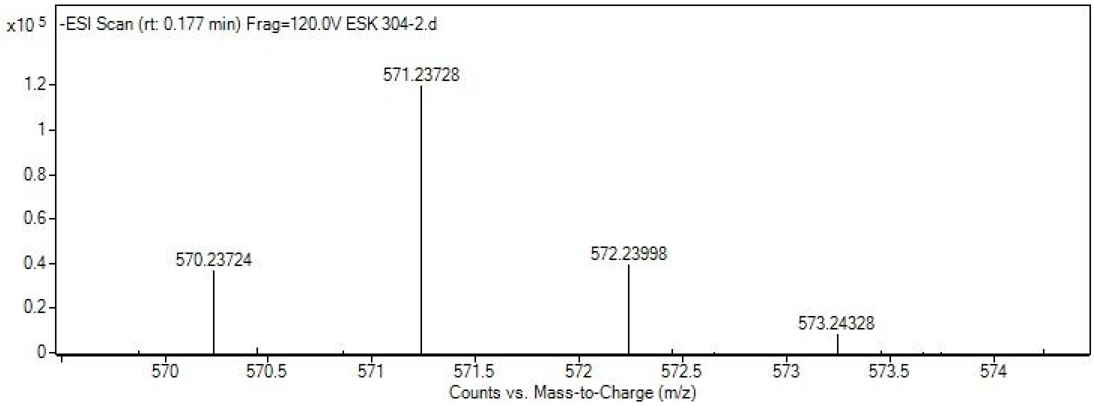

Figure S32. HRMS spectrum of **3aa$_2$**.
